# Allometry in limb regeneration and scale-invariant patterning as the basis of normal morphogenesis from different sizes of blastemas

**DOI:** 10.1242/dev.202697

**Published:** 2024-11-08

**Authors:** Saya Furukawa, Sakiya Yamamoto, Ayaka Ohashi, Yoshihiro Morishita, Akira Satoh

**Affiliations:** ^1^ Graduate School of Environmental, Life, Nature Science and Technology, Okayama University, Okayama 700-8530, Japan; ^2^ Laboratory for Developmental Morphogeometry, RIKEN Center for Biosystems Dynamics Research, Kobe 650-0047, Japan; ^3^ Research Core for Interdisciplinary Sciences (RCIS), Okayama University, Okayama 700-8530, Japan

**Keywords:** Limb regeneration, Axolotl, *Shh*, *Fgf8*, Blastema, Allometric scaling

## Abstract

Axolotl (*Ambystoma mexicanum*) limb regeneration begins with blastemas of various sizes, in contrast to the limb developmental process. Despite this size variation, normal limb morphology, consistent with a limb stump size, is regenerated. This outcome suggests the existence of underlying scale-invariant mechanisms. To identify such mechanisms, we examined the allometric relationships between blastema size, and *Sonic Hedgehog* (*Shh*) and *Fibroblast Growth Factor 8* (*Fgf8*) expression patterns against limb stump size. We found that all factors showed allometric rather than isometric scaling; specifically, their relative sizes decrease with an increase in limb stump size. However, the ratio of Shh/Fgf8 signaling dominant region was nearly constant, independent of blastema/body size. Furthermore, the relative spatial patterns of cell density and proliferation activity, and the relative position of first digit formation were scale invariant in the summed Shh/Fgf8 crosstalk region. This scale-invariant nature may underlie the morphogenesis of normal limbs from different sizes of blastemas.

## Teaser

**Highlighted Article:** Axolotl limb regeneration demonstrates scale-invariant patterning and allometric scaling, ensuring consistent limb morphogenesis from varying blastema sizes through coordinated Shh and Fgf8 signaling.

## INTRODUCTION

Urodele amphibians have outstanding regenerative capacity and are capable of regenerating multiple organs throughout their lives (Goss, 1969a). Limb regeneration, in particular, has a long research history as a representative example of regeneration. When limbs are amputated, regeneration-specific early events, such as wound healing, de-differentiation and initial blastema formation occur. In the subsequent morphogenesis phase, morphological changes and gene expression profiles show high similarity to limb development, and, therefore, regeneration is thought to recapitulate development (Muneoka and Bryant, 1982). For example, secreted factors such as *Shh* and *Fgf8* are essential for spatial patterning and cell proliferation, even in both limb development and limb regeneration (Torok et al., 1999; Nacu et al., 2016; Purushothaman et al., 2022). *Shh* is expressed in the posterior region of the blastema during limb regeneration, as it is expressed in the developing limb buds of amniotes and axolotls (Torok et al., 1999; Nacu et al., 2016; Purushothaman et al., 2019). However, *Fgf8* expression patterns in developing limb buds are significantly different between amniotes and urodele amphibians (Han et al., 2001; Nacu et al., 2016; Purushothaman et al., 2019). In the limb buds of amniotes, *Fgf8* is expressed in an ectodermal region called the apical ectodermal ridge, whereas *Fgf8* is expressed in the anterior mesenchyme in axolotl limb buds. Although the *Fgf8* expression domain is different, a recent study suggests that the interaction between *Shh* and *Fgf8* in a developing limb bud is established in axolotls in the same way as it is in amniotes (Purushothaman et al., 2019). In axolotls, it has further been suggested that the Shh/Fgf8 feedback loop contributes to anteroposterior axis formation (Nacu et al., 2016). In the case of axolotl limb regeneration, the unique *Fgf8* expression pattern and interaction between *Shh* and *Fgf8* are recapitulated in a regenerating blastema, highlighting the similarities between limb development and limb regeneration processes.

On the other hand, developmental and regenerative processes differ greatly in terms of initial size during morphogenesis. Limb development begins from a bud of approximately the same size within each species, whereas the initial sizes of the limb regeneration process can vary widely. As axolotls continue to grow permanently after development (Vieira et al., 2019), the initial size in the regenerative process is strongly influenced by the point in the life course at which limb damage occurs. Nevertheless, normal limb morphology is regenerated from different sizes of stump limbs. The mechanisms behind this are largely unknown.

To address this issue, we began by examining the allometric relationship between blastema size and gene expression patterns against limb stump/body size. Allometry, or allometric scaling, is when the relationship between the size of a tissue/organ of interest (i.e. a trait) depends on the size of a reference structure (e.g. head-to-tail length). This is mathematically expressed as *y=*k*x*^α^ or log(*y*)=α×log(*x*)+log(k), where *x* and *y* are the sizes of the reference and trait, respectively (Huxley, 1924, 1932; Huxley and Teissier, 1936). When α=1, it means that the trait changes isometrically or linearly depending on body size, and when α≠1, it means that the trait and reference sizes change at different rates. In the latter case, when α<1, the relative size of the trait to the reference is smaller as the reference size increases. Our analysis suggests that the size of the blastema scales allometrically with limb stump size (α<1). This means that blastema size is not completely proportional to limb stump size, although fully regenerated limb sizes match stump limbs after sufficient time (Wells et al., 2021). In contrast, the ratio of dominant regions of the Shh signal (representing the posterior signal) to that of the Fgf8 signal (representing the anterior signal) is nearly constant, independent of blastema or body size. Consistent with this scale-invariant chemical patterning, the relative spatial patterns of cell density and proliferation, and the relative position at which the first digit emerges are also scale invariant. We think that this scale invariance in patterning and growth is one of the basic mechanisms that allow consistent regeneration from an amputated limb or blastema, even if its size is different among each individual. Finally, we propose a model to consistently explain the obtained results.

## RESULTS

### Scaling of blastema size with animal and limb sizes

**Fig. 1.**
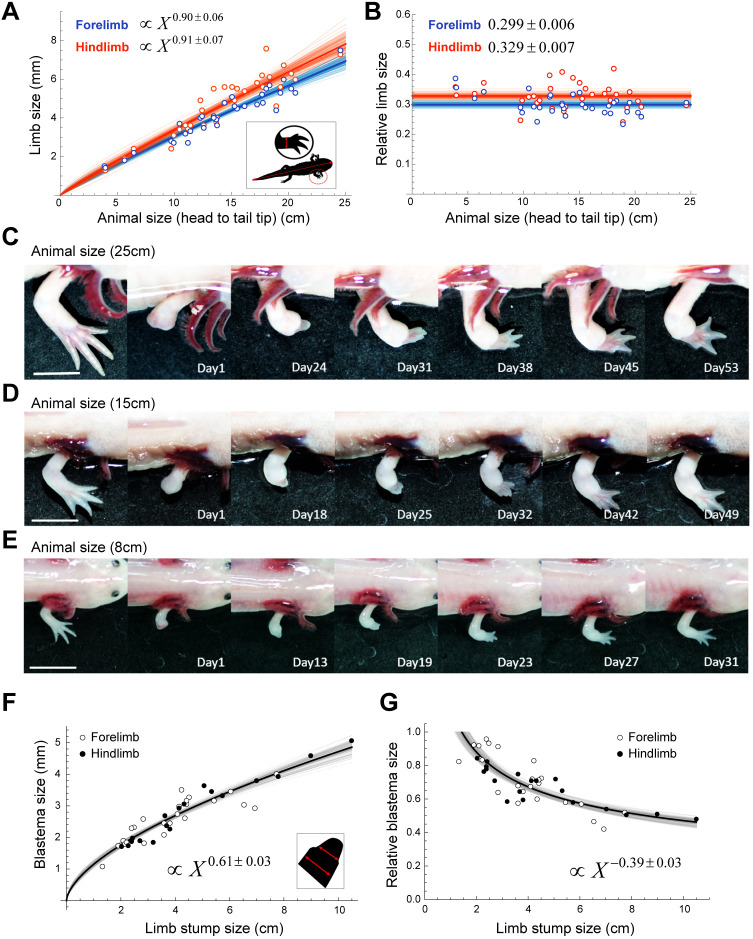
**Limb regeneration for animals of different sizes and allometric relashionships.** (A) The nearly isometric relationship between limb and animal size. Antero-posterior (A-P) limb widths were measured at the zeugopodial region (*n*=33). Allometric equations: Y=0.38X^0.90^ for forelimb and Y=0.41X^0.91^ for hindlimb. Using bootstrap samples, the standard deviation (s.d.) for the estimate of the exponent was also assessed: s.d.=0.06 for forelimb; 0.07 for hindlimb. (B) The limb size relative to animal size. Regression was performed on a constant function. s.d.=0.006 for forelimb; 0.007 for hindlimb. (C-E) Time course of the limb regeneration process for various sizes of animals. Scale bars: 1 cm. (F) The allometric relationship between blastema and limb stump size. The blastema size was evaluated by the A-P width at its proximal boundary. Owing to substantial overlap between forelimb and hindlimb data, both datasets were combined and regressed using a single allometric equation: Y=1.2X^0.61^. The s.d. of the exponent is 0.03. Forelimb, *n*=24; hindlimb, *n*=19. (G) The blastema size relative to the limb stump size: Y=1.2X^−0.39^.

To identify the scaling relationship between whole-animal size and individual organ sizes, we first quantified the relationship between animal size (defined as the anteroposterior distance from head to tail) and anterior-posterior (A-P) limb width in axolotls. The regression on an allometric formula widely used in the morphometrics field (*y=*κ*x*^α^) shows that limb/body size has an approximately linear relationship (α=0.90±0.06 for forelimb; α=0.91±0.07 for hindlimb), indicating that the relative limb width is almost constant, regardless of animal size (Fig. 1A,B, Fig. S1A: double logarithmic graph). Upon limb injury, axolotls can regenerate the limb completely, regardless of body size (Fig. 1C-E). Although the correlation between animal and blastema sizes may be intuitively apparent, little quantitative information is available. Thus, we also measured the blastema size (defined as its A-P width on the bottom plane blastemas) and the limb stump size (defined as its A-P width, which is nearly proportional to body size) to clarify their relationship (Fig. 1F,G, Fig. S1B). As the time required for regeneration strongly depends on body size, the regeneration stages between individuals were aligned based on the shape of the blastema (see Materials and Methods). The result showed that blastema and limb stump sizes have a clear scaling relationship. However, unlike the relationship between animal and limb sizes, the exponent was not close to 1 (α=0.61±0.03, Fig. 1A,B,F,G), meaning the relative size of blastema to limb stump size is smaller in larger animals (Fig. 1G). Thus, these quantifications demonstrated that there is a fixed, not isometric but allometric, scaling relationship between blastema size, and body and limb sizes.

### Clear scaling relationship between *Shh* expression and blastema size

**Fig. 2.**
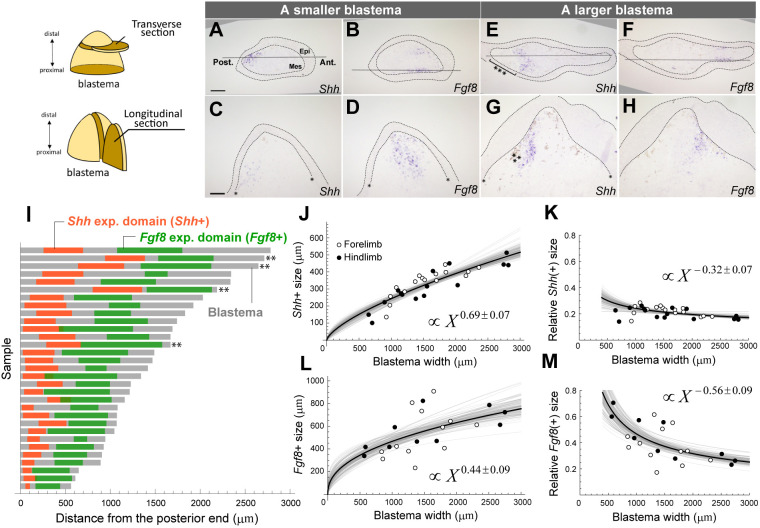
**Allometry of *Shh*(+) and *Fgf8*(+) sizes in blastemas.** (A-H) *Shh* or *Fgf8* expression was visualized by *in situ* hybridization in smaller blastemas (A-D) and larger blastemas (E-H). The dotted lines indicate the boundary of the blastema epithelium. Epi, epithelium; Mes, blastema mesenchyme. The single asterisks in C, D and G indicate the discontinuity in dermal collagen, i.e. the amputation site. The solid horizontal lines in A, B, E and F indicate the approximate position of the longitudinal sections. The triple asterisks in E and G indicate the gap between *Shh*(+) and the posterior epithelium. Scale bars: 200 µm. (I) The average widths of *Shh*(+) and *Fgf8*(+) collected from two or three pairs of adjacent sections for each identical blastema are shown in orange and green, respectively (*n*=31). Gray lines indicate the width of the blastema mesenchyme along the anteroposterior axis. The double asterisks indicate the samples with clear anterior shifts in gene expression patterns. (J,L) The allometric relationship between the size of *Shh*(+) or *Fgf8*(+) and the size of blastema mesenchyme. Y=2.1X^0.69^ for *Shh*(+) and Y=23X^0.44^ for *Fgf8*(+). The s.d. of the exponents: 0.07 for *Shh*(+) and 0.09 for *Fgf8*(+). *Shh*(+) forelimb, *n*=16; hindlimb, *n*=15. *Fgf8*(+) forelimb, *n*=12; hindlimb, *n*=10. (K,M) The sizes of *Shh*(+) and *Fgf8*(+) relative to blastema size: Y=2.1X^−0.32^ for *Shh*(+) and Y=23X^−0.56^ for *Fgf8*(+). The images in A, B, E and F have been rotated to make the A-P axis horizontal. The blank regions caused by the rotation are filled in gray.

*Shh* and *Fgf8* play essential roles in limb patterning and growth in both amniotes and urodele amphibians (Roy and Gardiner, 2002; Purushothaman et al., 2019, 2022). To understand how gene expression patterns vary based on blastema size, we investigated *Shh* and *Fgf8* expression patterns within various sizes of blastemas. We quantitatively compared the positions and sizes of *Shh*/*Fgf8* expression domains [hereafter denoted as *Shh*(+)/*Fgf8*(+)] after projecting them onto the A-P axis on the transverse section (Fig. 2A-I, Figs S2 and S3, see Materials and Methods). As previously reported (Torok et al., 1999), *Shh* was usually expressed in the posterior mesenchyme of a blastema, but in some larger blastemas, we observed anterior shifts of the *Shh*(+) away from the posterior-most end (i.e. immediately beneath the posterior epithelium) (Fig. 2E,G, triple asterisks, Fig. 2I). The reason for this anterior shift is unknown but may be related to the spatial heterogeneity of cell supply to the blastema, depending on how the stump tissues are damaged. *Fgf8* was expressed in the anterior mesenchyme, divided into dorsal and ventral regions close to the epithelium (Fig. 2B,F, Fig. S3, Movies 1 and 2). In the sample with the anterior shift of *Shh*(+), *Fgf8*(+) also shifted anteriorly in tandem (Fig. 2I, double asterisks). This observation suggests an interaction between *Shh* and *Fgf8* signaling, which we will examine later.

We also regressed the size of *Shh*(+)/*Fgf8*(+) on the allometric equation against blastema size (Fig. 2J,L, Fig. S1C,D). The size of *Shh*(+) fit the allometry equation very well with a little variance. The exponent was 0.69±0.07, which is not far from 1 (Fig. 2J). In fact, the size of *Shh*(+) relative to blastema size was nearly constant (at least within our focal range), with *Shh*(+) accounting for approximately 21% of blastema size (see the shallow slope in Fig. 2K). The size of *Fgf8*(+) showed a positive correlation with blastema size, although the variance was larger. The allometric exponent was much smaller than 1 (0.44±0.09), and its relative size to the blastema becomes clearly smaller as blastema size increases (Fig. 2L,M). In summary, the *Shh*(+) size reflects blastema size well and scales near-linearly with it, suggesting that this may be key for normal patterning and/or growth within blastemas of various sizes.

### Blastema size-independent patterning within the crosstalk region of Shh/Fgf8 signaling

**Fig. 3.**
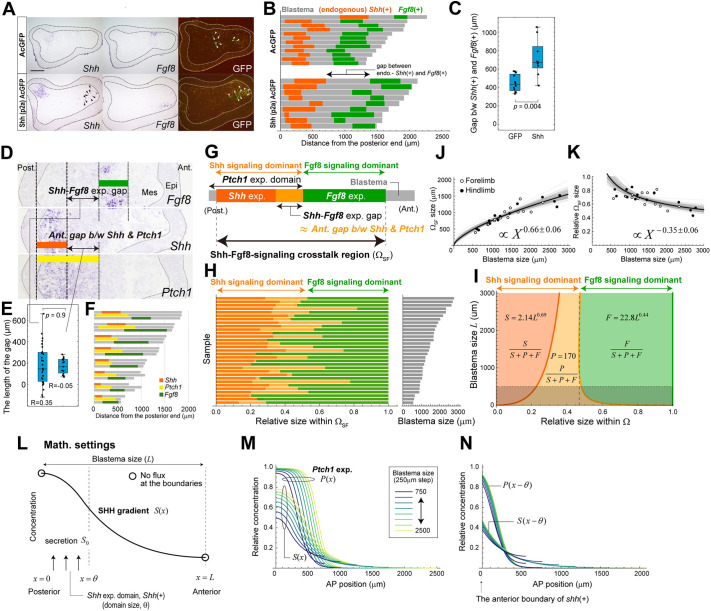
**Blastema size-independent patterning within the crosstalk region of Shh and Fgf8 signaling.** (A) The vectors pCS2-AcGFP or pCS2-Shh-p2a-AcGFP were electroporated into the blastema mesenchyme. The area surrounded by arrowheads indicates the area with pCS2-AcGFP or pCS2-Shh-p2a-AcGFP signal. The dotted lines indicate the boundary of the blastema epithelium. Scale bar: 400 µm. (B) The endogenous *Shh*(+) and *Fgf8*(+) widths are shown in orange and green, respectively. Gray lines indicate the width of the blastema mesenchyme along the A-P axis. (C) A box plot showing the gap between endogenous *Shh*(+) and *Fgf8*(+). pCS2-AcGFP introduction, *n*=12; pCS2-Shh-p2a-AcGFP introduction, *n*=9. Statistical analysis was performed using an unpaired two-tailed Student's *t*-test. (D,E) The A-P distance of the expression gap between *Shh* and *Fgf8* (left, *n*=31), and the anterior gap size between *Shh* and *Ptch1* (right, *n*=15). Epi, epithelium; Mes, blastema mesenchyme. R=correlation coefficient. (F) The *Shh*(+), *Ptch1*(+) and *Fgf8*(+) widths are shown in orange, yellow and green, respectively (see Materials and Methods). Gray lines indicate the sizes of blastema mesenchyme along the A-P axis. *n*=8. (G,H) The key quantities and their measurement data. We refer to the range from the posterior end of *Shh*(+) to the anterior end of *Fgf8*(+) as the Shh/Fgf8 signaling crosstalk region (denoted as Ω_SF_), the range from the posterior end of *Shh*(+) to the anterior end of *Ptch1*(+) as the Shh signaling dominant region (composed of orange and yellow regions), and *Fgf8*(+) as the Fgf8 signaling dominant region (green). The left bar graph in H shows the relative sizes of Shh/Fgf8 signaling dominant regions within the Ω_SF_. The right graph shows the absolute sizes of blastemas for each sample (*n*=31). (I) The dependence of the relative sizes of *Shh*(+) (orange), *Ptch1*(+) (orange and yellow) and *Fgf8*(+) (green) within the Ω_SF_ on the blastema size, calculated from the allometric equations shown in Fig. 2J,L. S, *Shh*(+) size; *P*, the *Shh*-*Fgf8* expression gap (≒ anterior gap between *Shh* and *Ptch1*); *F*, *Fgf8*(+) size; L, blastema size. Black shading indicates that the calculation for blastema sizes below 500 µm is based on extrapolation using those equations (i.e. no measurement data for blastemas <500 µm). (J) Allometric relationship between the size of the Ω_SF_ and the size of blastema mesenchyme: Y=8.2X^0.66^. s.d. of the exponent: 0.06. Forelimb, *n*=15; hindlimb, *n*=13. (K) The size of the Ω_SF_ relative to that of blastema: Y=8.2X^−0.35^. (L-N) Calculation of SHH diffusion and *Ptch1* expression patterns using a mathematical model (see Materials and Methods for details). (L) Mathematical settings. (M) The spatial profiles of SHH concentration and *Ptch1* expression levels within blastemas of different sizes. (N) The results obtained by re-plotting the profiles in M with the anterior boundary of *Shh*(+) as the reference. The boxes represent the interquartile range (IQR), where the central line indicates the median. The whiskers extend to the minimum and maximum values within the data.

As mentioned above, the positions of *Shh*(+) and *Fgf8*(+) can change in tandem. Previous studies reported the crosstalk between Shh and Fgf8 signaling during limb development and/or regeneration, and mutual activation and/or inhibition pathways are known to exist and function in a complex manner (Kraus et al., 2001; Nacu et al., 2016; Matsubara et al., 2017; Purushothaman et al., 2019, 2022). To confirm the interdependent arrangement of *Shh*(+) and *Fgf8*(+), *Shh* was expressed ectopically by electroporation around the center of the blastema (Fig. 3A). As a result, *Fgf8*(+) shifted to the anterior side more than usual, causing expansion of the gap between endogenous *Shh*(+) and *Fgf8*(+) (Fig. 3B,C). This demonstrates that the expression patterns of *Shh* and *Fgf8* are not independent. It should be noted that although we are interested in the more-quantitative regulatory relationship between *Shh* and *Fgf8* expression, it was difficult to evaluate this in the current experimental system. In the electroporation method, it is difficult to precisely control the location and level of expression induction, which made it challenging to perform repeatable experiments with reproducible induced patterns. As an exploratory trial, we examined the relationship between the total area of GFP(+) regions and the gap between endogenous *Shh*(+) and *Fgf8*(+), and we were unable to observe a clear correlation between them (Fig. S4), although the mean gap size in the case of *Shh* induction is significantly larger compared with that of the control case as seen in Fig. 3C. Clarifying the quantitative regulatory relationship between the spatial expression patterns of *Shh* and *Fgf8* will be an important issue for future research.

As mentioned above, in many individuals, there is a spatial gap along the A-P axis between *Shh*(+) and *Fgf8*(+) (Fig. 3D, compare the upper two images). The size of the gap was on average 170 µm and it did not correlate well with blastema size (R=0.35), although the variance was relatively large between individuals (Fig. 3E, left box plot). *Patched 1* (*Ptch1*), which encodes a receptor for SHH and is used as a SHH indicator (Marigo et al., 1996; Lewis et al., 2001), is expressed in the blastema, covering *Shh*(+) (Fig. 3D,F, Fig. S5). In particular, on the anterior side, the gap between *Shh*(+) and the *Ptch1* expression domain [denoted by *Ptch1*(+)] was generally constant and approximately 170 µm on average, which was comparable with the spatial gap between *Shh*(+) and *Fgf8*(+), regardless of blastema size (Fig. 3E). In short, the posterior end of *Fgf8*(+) was almost identical to the anterior end of *Ptch1*(+) (Fig. 3D-G), indicating that the blastema could be divided into the Shh signaling dominant region [the region between the posterior end of *Shh*(+) and the anterior end of *Ptch1*(+)] and the Fgf8 signaling dominant region [*Fgf8*(+)].

As previously mentioned, the size of *Shh*(+) scales almost linearly with blastema size, whereas the relative size of *Fgf8*(+) becomes smaller as blastema size becomes larger. Interestingly, we found that the proportions of the Shh/Fgf8 signaling dominant regions are almost scale invariant (i.e. independent of blastema size) within their crosstalk region (denoted as Ω_SF_, see also Fig. 3G), defined as the range from the posterior end of *Shh*(+) to the anterior end of *Fgf8*(+) (Fig. 3H, Fig. S6). More precisely, calculation using the allometry equations for *Shh*(+) and *Fgf8*(+) against the blastema size quantified above (Fig. 2J,L) showed that each of the Shh and Fgf8 signaling dominant regions have an almost 50% proportion (specifically, the Shh signaling dominant region occupies 47% and the Fgf8 signaling dominant region occupies 53%) within Ω_SF_ for any blastema size we examined (within the range of 500 µm to 3000 µm; Fig. 3I). Intuitively, this can be understood as follows. Although *Shh*(+) size scales almost linearly with blastema size, the diffusion range of SHH protein secreted from *Shh*(+) does not scale with blastema size because the physical constants, diffusion and degradation coefficients are likely invariant. In fact, the distance between the anterior end of *Ptch1*(+) and the anterior end of *Shh*(+) is independent of blastema size (R=−0.05) (Fig. 3E, right box plot). This independence is further supported by solving the diffusion equation with biologically plausible settings and parameter values (Fig. 3L-N, see Materials and Methods). Even *Shh*(+) size linearly scales with blastema size, the distance between both anterior ends of *Shh*(+) and *Ptch1*(+) was almost constant. As the SHH diffusion range is constant as above, the relative size of the Shh signaling dominant region [as defined by the distance from the *Shh*(+) posterior end to the *Ptch1*(+) anterior end] to blastema size decreases with the increase in blastema size. The relative size of *Fgf8*(+) also becomes smaller within larger blastemas. As demonstrated above, the expression of *Fgf8* depends on the Shh signal. In the context of limb development, it is known that SHH activates *Fgf8* expression. However, considering the gap between *Shh*(+) and *Fgf8*(+), it is likely that there exists an inhibitory pathway, whether indirect or direct, in addition to the activation pathway. To quantitatively explain the allometric relationship between *Fgf8*(+) and blastema sizes (Fig. 2L,M), it would be necessary to elucidate the detailed dose-response relationship for *Fgf8* expression induced by the Shh signal. In any case, within the Shh/Fgf8 crosstalk region Ω_SF_, the blastema size-invariant proportions are achieved by the well-balanced dependence of the sizes of Shh and Fgf8 signaling dominant regions on blastema size (Fig. 3I). In contrast, the relative size of the crosstalk region Ω_SF_ itself decreases with the increase in blastema size, i.e. allometrically scaling with a relatively smaller exponent (α=0.66±0.06) (Fig. 3J,K, Fig. S1E). We should note that the allometric equation we quantified is a regression on data within a specific range of blastema sizes (500-3000 µm). Therefore, it remains uncertain whether the same equation holds true for blastemas smaller or larger than this range.

Taken together, within the Shh/Fgf8 crosstalk region Ω_SF_, the proportions of Shh and Fgf8 signaling dominant regions are invariant, regardless of blastema size (for blastemas of 500-3000 µm), suggesting that this invariant proportion is one of the key mechanisms for normal regeneration. In the following sections, to clarify this importance, we quantify the cell density and proliferation distributions and the chondrogenic patterns in blastemas of various sizes.

### Scale-invariant patterns of cell density and proliferative activity

**Fig. 4.**
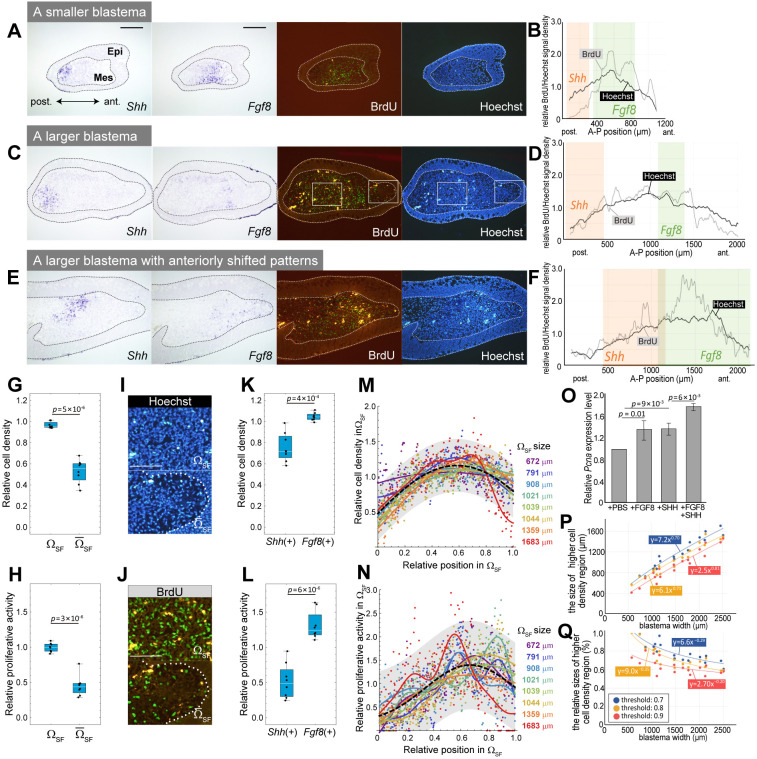
**Scale-invariant patterns of cell density and proliferative activity.** (A,C,E) *Shh* and *Fgf8* expression was visualized by *in situ* hybridization. BrdU incorporation was visualized using immunofluorescence, and nuclei were visualized using Hoechst. Dotted lines indicate the boundary of the blastema epithelium. Scale bar: 400 µm. Epi, epithelium; Mes, blastema mesenchyme. (B,D,F) Spatial profiles of cell density (Hoechst signal, black) and proliferative activity (BrdU signal, gray), quantified from image data (see Materials and Methods for details). *Shh*(+) and *Fgf8*(+) are shown as orange and green, respectively. (G,H) The mean values of relative Hoechst (G) and BrdU (H) signals within or outside the Ω_SF_ (*n*=8). Statistical analysis was performed using an unpaired two-tailed Student's *t*-test. (I,J) High-magnification image of the marked region in C. Each upper image is in the Ω_SF_; the lower is outside the Ω_SF_ (=Ω̅_SF_). Scale bars: 400 µm. (K,L) The mean values of relative Hoechst (K) and BrdU (L) signals within *Shh*(+) or *Fgf8*(+) (*n*=8). The boxes represent the interquartile range (IQR), where the central line indicates the median. The whiskers extend to the minimum and maximum values within the data. (M,N) Normalized patterns of cell density (M) and proliferative activity (N) within the Ω_SF_. Colored lines show cell density or cell proliferation activity for each sample. The black dashed lines show the unimodal functions fitting all eight samples. The gray-colored region shows the 95% confidence interval. (O) The relative *Pcna* expression level of cultured blastema mesenchymal cells with added recombinant proteins was measured using quantitative reverse transcription polymerase chain reaction. Statistical analysis was performed using a one-way analysis of variance with Ryan's post-hoc test. Data are mean±s.d. (P) The sizes of regions with cell densities above certain thresholds were measured and regressed on the allometric equation (*n*=18). (Q) A scatter plot showing the relationship between the occupation ratio of the region with higher cell density and blastema size.

Next, we examined the spatial patterns of cell density and proliferative activity for individuals with different sizes of blastema, paying particular attention to cells within the Shh/Fgf8 crosstalk region Ω_SF_. To quantify cell density and cell proliferation activity, we created spatial profiles of Hoechst signals and BrdU incorporation, respectively, based on image data (Fig. 4A-F, see Materials and Methods). As signal intensity varied across samples, we used median-normalized distributions for the comparison of cell density and proliferative activity among individuals with different sizes of blastema. In addition, they were superimposed on the images showing *Shh* and *Fgf8* expression patterns to examine the relationship with the patterning of those secreted molecules (Fig. 4B,D,F). Regardless of blastema size, cell density and proliferative activity were higher in the crosstalk region of Shh and Fgf8 signaling than outside this region (Fig. 4G-J). Within the crosstalk region, the values in *Fgf8*(+) were significantly higher than those in *Shh*(+) (Fig. 4K,L). Interestingly, the distributions of density and proliferation can shift within a blastema; in larger blastemas with anteriorly shifted *Shh*(+) and *Fgf8*(+), the region with higher density and proliferative activity was also anteriorly shifted (Fig. 4E,F). Furthermore, in individuals in which *Fgf8*(+) was anteriorly shifted by ectopic *Shh* expression around the center of a blastema, the area showing higher cell density and proliferative activity appeared to be expanded to the anterior region, where the mitotic activity was relatively lower in the normal blastema (Fig. S7V, Movies 3 and 4, *n*=4/5).

As demonstrated in the previous section, the relative size of the Shh/Fgf8 crosstalk region (Ω_SF_) decreases with the increase in blastema size, but the proportion of the Shh and Fgf8 signaling dominant regions within the Ω_SF_ is almost constant. To determine whether the spatial distributions of density and proliferative activity also show such scale invariance within the Ω_SF_, we compared their distributions normalized by the size of the Ω_SF_ among individuals (Fig. 4M,N). The results revealed that their relative patterns of cell density within the Ω_SF_ were reproducible across individuals, independent of the Ω_SF_ (or blastema) size (Fig. 4M). Cell density peaked around the central region of the Ω_SF_ (more precisely, a little anterior to the center, approximately 60% from the posterior end) in blastemas of all sizes we examined, which is thought to be more affected by both Shh and Fgf8 signaling. We further observed large variations in the distribution of cell proliferation among samples. However, the peak of cell proliferation activity still basically occurred a little anterior to the center. Notably, the peak position of the regression curve for the data from all samples closely matched that of the cell density distribution (the black broken curve in Fig. 4M,N). To confirm the effect of these two signals on mitotic activity, SHH and/or FGF8 were applied to cultured blastema mesenchymal cells (Fig. 4O). Application of SHH or FGF8 increased the levels of *Proliferating Cell Nuclear Antigen* (*Pcna*) compared with the control, which is consistent with a previous report (Purushothaman et al., 2019). When both were applied, the *Pcna* level further increased. This provides a possible explanation for the maximum cell density and proliferative activity around the area where the presence of both SHH and FGF8 is expected.

Finally, as with the Ω_SF_ size, we observed that the size of the region with high density did not scale at the same rate (i.e. linearly) with blastema size (Fig. 4P, Fig. S1F). Plotting the relative sizes of regions with cell densities above a certain threshold value against blastema size revealed a monotonic decrease with the increase in blastema size (Fig. 4Q, Fig. S1G). In summary, the promotion of cell proliferation by Shh and Fgf8 signaling is considered to determine the location of regions with high cell density and proliferative activity within a blastema. Owing to the blastema size-independent proportion of Shh and Fgf8 signaling dominant regions within the Ω_SF_, the spatial patterns of cell density and proliferative activity in the crosstalk region Ω_SF_ are also invariant in scale.

### Reproducibility of the relative position of first digit formation

**Fig. 5.**
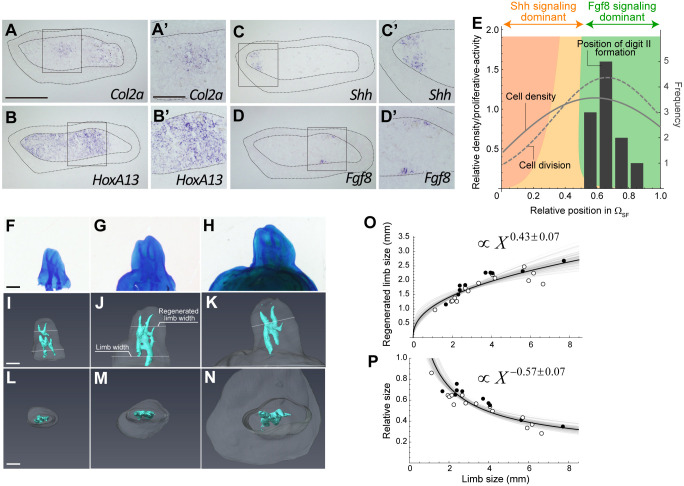
**Reproducibility of the relative position of first digit formation.** (A-D) *Col2a*, *Hoxa13*, *Shh* and *Fgf8* were visualized using *in situ* hybridization on the adjacent sections. Dotted lines indicate the boundary of the blastema epithelium. High-magnification images of the marked region are shown in the right panels (A′-D′). Scale bars: 500 µm in A; 200 µm in A′. (E) The relative position of the first forming digit (digit II) within the Ω_SF_ (black histogram) (*n*=11). The average profiles of cell density (gray, solid) and division (gray, dashed) quantified in Fig. 4M,N are also shown. Background colors are applied from Fig. 3I; the vertical axis indicates the blastema size. (F-H) Alcian Blue (AB) staining images of regenerating limbs. Scale bar: 1 mm. (I-N) 3D reconstruction of AB-stained samples shown in F-H. Light blue indicates cartilage. Gray regions show regenerating limbs. (I-K) Dorsal views. (L-N) Top views. Scale bars: 1 mm. (O) The allometric relationship between the sizes of regenerating limbs and stump-limbs. Y=1.1X^0.43^, s.d.=0.07. Forelimb, *n*=14; hindlimb, *n*=10. (P) The sizes of regenerating limbs relative to those of stump limbs: Y=1.1X^−0.57^.

To understand how the distribution of cell density within the Shh/Fgf8 crosstalk region (Ω_SF_) is involved in morphogenesis during limb regeneration, we examined its relevance to the pattern of digit chondrogenic differentiation, which is a representative feature of limb morphogenesis. In previous studies using chick mesenchymal cell culture systems, higher cell density was reported to promote chondrogenic differentiation (Matsutani and Kuroda, 1978; Ide, 1990). In particular, we focused on the formation of digit II, which is the first to form (Nye et al., 2003). *Collagen Type II Alpha* (*Col2a*) was used as an early marker for the formation of digit II; *Col2a* expression can be detected by *in situ* hybridization at a slightly later stage (1-2 days later) than the late bud stage used in the above analyses (Fig. 5A). The expression patterns of *HoxA13* as a marker for the prospective autopod region, and *Shh* and *Fgf8* to define Ω_SF_, were also visualized by *in situ* hybridization using adjacent tissue sections (Fig. 5B-D). It should be noted that *Shh* and *Fgf8* expression begins to decline at this time but expression of both genes could still be detected.

*Col2a* expression begins within the Ω_SF_. When normalized by Ω_SF_ size, the relative position of the center of *Col2a* expression coincided well with the location of the highest cell density, with a maximum frequency of digit II formation among samples around 60% from the posterior end of the Ω_SF_ (Fig. 5E). Variation in the relative position of *Col2a* expression (precisely, using the value of the coefficient of variation) was greater when normalized by blastema size than when normalized by the Ω_SF_ (the former: 0.158; the latter: 0.126). This outcome supports the idea that the position of digit II formation is regulated such that it scales within the Ω_SF_ regardless of blastema size. Although it is difficult to directly prove causality, it is suggested that increasing cell density in the Ω_SF_ beforehand leads to digit II formation, as maximal density is already observed in its presumptive region before apparent digit chondrogenesis begins. We also examined how ectopic *Shh* expression affects the formation of digit II (Fig. S8). As a result, we found that the induction of ectopic *Shh* expression delayed the timing of digit differentiation, probably because Shh signaling maintains mesenchymal cells in an undifferentiated state. In the induction using the electroporation method, it is difficult to precisely control the location and amount of the ectopic expression, which likely caused variability in the degree of differentiation delay between samples. This made it difficult to determine the timing of the first digit formation and to quantitatively evaluate the location where the formation begins.

Finally, we investigated the allometry of the regenerating limb size (L_reg_limb_) during subsequent digit cartilage formation against limb stump sizes (L_stump_), in proportion to individual body size. The secondary growth after finishing basic anatomical patterning, including digit chondrogenesis, allows a regenerating limb to grow to approximately the same size as the limb stump size after a sufficient amount of time. Thus, the allometry between L_reg_limb_ and L_stump_ was quantified by measuring the size of the regenerating limb at the stage before this secondary growth, when at least part of the digit cartilage, including digit II, was stained using Alcian Blue (Fig. 5F-N) (e.g. several days after the late bud stage). As a result, we obtained the relationship L_reg_limb_ ∝ (L_stump_)^0.43^ (Fig. 5O,P, Fig. S1H). The value of the exponent is much smaller than 1, meaning that as limb and/or individual size increases, the relative size of the regenerating limb (before secondary growth) decreases. This indicates that the early regenerating limb cannot perfectly (i.e. linearly) scale with body size.

## DISCUSSION

### Scale-invariant patterning that allows normal limb regeneration in axolotls

**Fig. 6.**
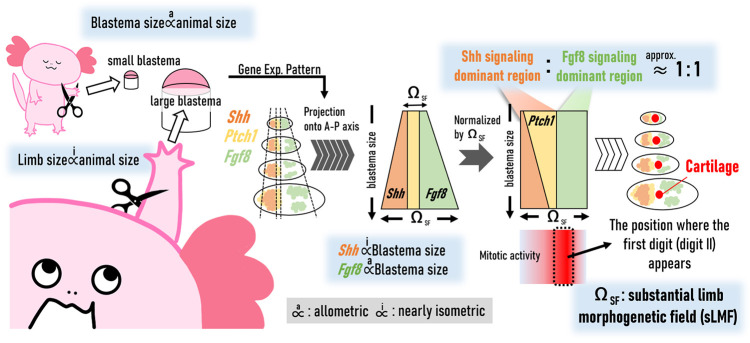
**Scale-invariant patterns of gene expression, cell density and proliferation within the Ω_SF_ for normal limb regeneration in various sizes of individuals.** Limb size is almost proportional to animal size. Blastema size also monotonically depends on limb size (not isometrically, but allometrically), suggesting that there should be mechanisms to achieve normal patterning and morphogenesis in regeneration fields of different sizes. The *Shh*(+) (*Shh* expression domain) scales nearly isometrically with blastema size, whereas the *Fgf8*(+) (*Fgf8* expression domain) shows allometric scaling. Importantly, the proportions of Shh and Fgf8 signaling dominant regions within the Ω_SF_, which is defined as a region from the posterior end of the *Shh*(+) to the anterior end of the *Fgf8*(+), are scale invariant; both regions occupy about half of the Ω_SF_. Furthermore, the spatial patterns of cell density and proliferation, rescaled by the size of Ω_SF_, are also conserved regardless of blastema and limb stump size. Finally, the first digit to form (digit II) appears at a fixed relative position, with the highest cell density, within Ω_SF_. Thus, this scale-invariant patterning within the Ω_SF_ is considered to provide a basis for normal limb regeneration in individuals of different sizes. In this sense, the Ω_SF_ can be regarded as a substantial limb morphogenetic field (sLMF).

We have shown that within the Ω_SF_ (i.e. the Shh/Fgf8 crosstalk region), the A-P proportion of Shh and Fgf8 signaling dominant regions (Fig. 3G-I), the relative spatial pattern of cell density and proliferation (Fig. 4M,N), and the relative position of the first digit (digit II) formation (Fig. 5E) remain almost constant regardless of blastema or body size. This scale-invariant patterning is considered to be an important underlying mechanism that allows normal morphogenesis during axolotl limb regeneration from different sizes of blastemas. Consequently, the region Ω_SF_, where such scale-invariant behaviors are observed, may be regarded as a substantial limb morphogenetic field (sLMF) within a blastema (Fig. 6). Furthermore, the absolute location of the Ω_SF_ does not appear important. In fact, even when the Ω_SF_ is anteriorly shifted, which is likely to occur in a larger blastema, the scale-invariant patterning and growth within the Ω_SF_ are still conserved and consistent with those in a blastema without the anterior shift (Fig. 4E,F). As stated earlier, it should be noted that this scale invariance holds at least within blastemas ranging from 500 µm to 3000 µm; it is not guaranteed that the allometry equation holds for smaller blastemas. For example, our equation suggests that as the blastema size approaches 0, the relative size of *Ptch1*(+) within the Ω_SF_ becomes excessively broad (Fig. 3I), which is unrealistic. Additionally, obtaining sufficient data to understand the patterning within blastemas larger than 3000 µm would require a considerable amount of time and be quite challenging. Furthermore, the above-mentioned allometry and scale invariance were derived from regeneration data after lower arm amputations. As the blastema morphology and proliferation rate within lower arms are known to be different from the regeneration after upper arm amputations (Goss, 1969b; Iten and Bryant, 1973), comparing these data with the current results would be an interesting point for future research.

### Scale variance of the Ω_SF_ size

In contrast to scale-invariant limb patterning within the Ω_SF_, the size of the Ω_SF_ itself allometrically scales with blastema size; thus, it is scale variant (Fig. 3J). The Ω_SF_ consists of three regions: (1) *Shh*(+); (2) the region from the anterior end of *Shh*(+) to the anterior end of *Ptch1*(+) [denoted as *Shh*(A)**-***Ptch1*(A)]; and (3) *Fgf8*(+). First, the size of *Shh*(+) relative to blastema size is nearly constant (Fig. 2K). The detailed molecular mechanism behind this is not known, but it may reflect a history of positional information within the limb before amputation. We have previously demonstrated that *Shh*-expressing cells only emerge from posteriorly derived cells (Iwata et al., 2020). In addition, more recently, it has been reported that *Hand2* expression serves as the posterior memory and also induces *Shh* re-expression during limb regeneration (Otsuki et al., 2023 preprint). Thus, it is very likely that the number of posterior-derived cells on the amputated plane affects *Shh*(+) sizes; larger limbs contain a greater number of posterior cells, resulting in larger *Shh*(+) sizes in proportion to blastema sizes. This explains why *Shh*(+) size scales almost linearly with blastema size. Second, *Shh*(A)-*Ptch1*(A), which is comparable with the gap between *Shh*(+) and *Fgf8*(+), was nearly constant in various sizes of a blastema. This would be due to the constant SHH diffusion range for any size of blastema. Our results further suggested that the SHH diffusion range from the anterior end of *Shh*(+) is not significantly affected by *Shh*(+) size, probably because of the physical constants for diffusion and degradation. Finally, the *Fgf8*(+) size is thought to be affected by Shh signaling. It has been reported that Shh signaling activates *Fgf8* expression (Nacu et al., 2016; Purushothaman et al., 2019). We also found that inducing ectopic *Shh* expression around the center of blastemas shifts the expression range of *Fgf8* more anteriorly. This result suggests that there is not only an activation pathway from *Shh* to *Fgf8*, but also a direct or indirect inhibitory pathway. The combination of these activation and inhibition pathways could explain how *Fgf8*(+) is regulated to a location that is not adjacent to *Shh*(+), but is also not far away from it. Furthermore, the constant SHH diffusion range may restrict the expansion of *Fgf8*(+) with an increase in blastema size, resulting in a decrease of the relative *Fgf8*(+) size as blastema size increases. It is also possible that signals other than Shh contribute to the regulation of *Fgf8* expression. For example, in the limb buds of amniotes, the feedback loop between *Fgf8* and *Fgf10* is established independently of Shh signaling (Ohuchi et al., 1997), and it has been reported that *Fgf10* is also expressed in the axolotl limb bud and blastema (Christensen et al., 2002). We should also note that *Fgf8*(+) sizes exhibit relatively large variability around their regression equation compared with *Shh*(+). It has been suggested that, during the regeneration process, anterior memory is easier to change than posterior memory, which is maintained within unamputated limbs by *Hand2* (Otsuki et al., 2023 preprint). This could explain why the size of *Fgf8*(+) follows the scaling law with greater variability than the size of *Shh*(+). Another possible explanation is the precision required to detect the posterior boundary of *Fgf8*(+). In this study, we determined the posterior boundary based on images from an *in situ* hybridization assay. In the future, with more quantitative data from techniques such as spatial transcriptomics, it might be possible to assess the variability in boundary positions. Taken together, the size of the Ω_SF_, i.e. the sum of the sizes of the above three regions, does not have a linear dependence on blastema size but does exhibit allometric scaling.

### Formation of a tiny limb during limb regeneration possibly due to the expansion limit of Ω_SF_

It is well known that after a sufficient amount of time, a regenerating limb grows until it is consistent with the limb stump size. However, as shown above, in the late stage blastema, the size of the Ω_SF_ in which morphogenesis occurs is allometrically dependent on blastema size: L_Ω_ ∝ (L_blastema_)^0.66^ (Fig. 3J). Furthermore, blastema size in this stage also scales allometrically with limb (stump) size: L_blastema_ ∝ (L_stump_)^0.61^ (Fig. 1F). Composing these two relationships yields L_Ω_ ∝ (L_blastema_)^0.66^ ∝ ((L_stump_)^0.61^)^0.66^ = (L_stump_)^0.40^; in other words, within larger individuals, the size of the morphogenetic field Ω_SF_ relative to limb size is much smaller. This exponent (α=0.40) is approximately maintained even when the subsequent digit cartilage patterning begins to occur: L_reg_limb_ ∝ (L_stump_)^0.43^. More precisely, the exponent value 0.40 is included in the 95% confidence interval (*P*=0.18) obtained from the data shown in Fig. 5O. After the basic anatomical patterning is completed, it has been reported that the regenerating limb grows to catch up with the limb stump size at a higher growth rate than that of intact limbs (Wells et al., 2021). A tiny limb may be formed at first during limb regeneration due to the expansion limit of the Ω_SF_, which is caused by the constant protein diffusion range. To achieve consistent limb morphogenesis, the Ω_SF_ must be of a size in which Shh and Fgf8 signaling can properly interact. In the future, it would be interesting to investigate how limb regeneration is affected when allometry is altered by changing the Ω_SF_ size or the proportion of the Shh and/or Fgf8 signaling dominant regions inside the Ω_SF_, e.g. by artificially altering the diffusivity of SHH using synthetic biology techniques (Toda et al., 2020).

### Positional variation of the Ω_SF_ due to the heterogeneity of cell supply to the blastema and the axolotl-specific *Fgf8* expression pattern

In this study, we found that the positions of the *Shh*(+) and *Fgf8*(+) show more variability in larger blastemas (Fig. 2I). In amniotes, it has been reported that *Shh* is expressed in the posterior margin of the developing limb bud (Riddle et al., 1993). However, in axolotl blastemas, *Shh*(+) was not always located in the posterior margin next to the epithelium (Fig. 2E,G,I). In limb development, the segregation of *Shh*(+) from the epithelium has not been reported to date and is characteristic of axolotl limb blastemas. Unlike in limb development, blastema cells are derived from fully differentiated tissues, such as the dermis (Muneoka et al., 1986; Hirata et al., 2010). We have previously demonstrated that Shh-expressing cells emerge only from posteriorly derived cells, suggesting that gene expression in blastemas is restricted by cell origin. A quantitative analysis of the contribution from dorsal, ventral, anterior and posterior tissues has not yet been carried out. However, it is very likely that the cell contribution from each location of the stump varies depending on where and how much tissue was damaged by limb amputation. Hence, the ratio of blastema cells derived from anterior and/or posterior tissues may be uneven in each blastema, causing variation in the position of the Ω_SF_. Moreover, mesenchymal *Fgf8* expression may also contribute to the Ω_SF_ positional variation and the segregation of *Shh* from the epithelium. In amniotes, it has been reported that the *Shh*(+) restriction to the posterior margin in limb buds is controlled by *dHand* (*Hand2*), *Tbx2*, *Gremlin1*, *Alx4* and *Fgf8* (Crossley et al., 1996; Robert and Lallemand, 2006; Farin et al., 2013; Matsubara et al., 2017). Among these, only *Fgf8* exhibits an apparent difference in expression pattern: *Fgf8* is expressed not in the epithelium but in the anterior mesenchyme of axolotl blastemas (Fig. 2B,D,F,H) (Nacu et al., 2016). In amniotes, *Fgf8* is expressed in an epidermal region called the apical ectodermal ridge and plays a role in the epithelial-mesenchymal signaling loop (Mariani et al., 2017). In axolotls, although the region where *Fgf8* is expressed is apparently different, it has been reported that *Shh* and *Fgf8* have a positive-feedback loop within the blastema mesenchyme (Nacu et al., 2016). As the signaling loop is confined to the mesenchymal region, *Shh*(+) would no longer need to be located immediately under the epithelium in axolotl blastemas. It is therefore likely that heterogeneity in the cell supply and axolotl-specific *Fgf8*(+) explain the shift in the position of Ω_SF_ and *Shh*(+) away from the epithelium, although more-detailed studies are needed.

## MATERIALS AND METHODS

### Animals and surgery

We used axolotls (*Ambystoma mexicanum*) with a nose-to-tail length of 4 to 25 cm, which we obtained from the private breeders and axolotl colony in the Hiroshima University. The axolotls were housed in aerated water at 22°C. Before all surgeries, axolotls were anesthetized using MS-222 (Sigma-Aldrich) for about 10 min (depending on the animal size). Limbs were amputated at the middle of the zeugopod. All animal use was approved by the Animal Care and Use Committee, Okayama University (licence 580 to A.S), and all animal experiments were conducted following the guidelines of Okayama University. We defined the late-bud stage blastema based on its appearance, which is characterized by dorsoventral flattening and a ratio of anterior-posterior to proximal-distal length of approximately 1:1.

### Sectioning, *in situ* hybridization, immunofluorescence and nuclear visualizing

Samples were fixed with 4% paraformaldehyde for 1 day at room temperature. After a 30% sucrose/PBS treatment for ∼12 h, they were then embedded in OCT compound (Sakura Finetek). Frozen sections with a thickness of 10μm were prepared using a Leica CM1850 cryostat. The sections were dried under an air dryer and kept at −80°C until use. RNA probes for *Fgf8, Shh, HoxA13* and *Col2a* were selected as previously described (Satoh et al., 2007; Makanae et al., 2014; Mitogawa et al., 2015; Iwata et al., 2020). The axolotl patched 1 (*Ptch1*) gene was newly isolated. The isolated sequences are from 1016-2989 of AMEX60DD201043057.6 in the axolotl OMICS site (https://www.axolotl-omics.org/). *In situ* hybridization was performed as previously reported (Mitogawa et al., 2015). Briefly, samples were treated with proteinase K (10 µg/ml) (Thermo Fisher) at room temperature for 20 min, and riboprobes were hybridized at 62.5°C. After hybridization, samples were washed with buffer 1 [50% formamide and 5× saline-sodium citrate (SSC)] twice for 20 min and buffer 2 (50% formamide and 2×SSC) three times for 20 min at 62.5°C. After samples were blocked with 0.5% blocking reagent (11096176001, Roche) in TBST, anti-digoxigenin-AP Fab fragments (11093274910, Roche) were added at 1:1000 dilution and incubated for 2 h at room temperature. Staining was carried out with NBT (148-01991, Wako-Fuji film) and BCIP (05643-11, Nakalai Tesque) in NTMT [100 mM NaCl, 0.1 M Tris (pH 9.5) and 0.1% Tween20]. For *in situ* hybridization and BrdU double staining, *in situ* hybridization was performed first, and immunostaining was subsequently performed. Immunofluorescence of the sections was performed as described in previous studies (Makanae et al., 2014). BrdU antigen retrieval was performed with 2 mol/l HCl for 30 min at room temperature. Anti-BrdU (G3G4, 1:300, DSHB) was used as the primary antibody and anti-mouse IgG Alexa Fluor 488 (A11017, 1:500, Invitrogen) was used as the secondary antibody. Immunofluorescence for GFP was performed on the adjacent other sections, thereby avoiding protein degradation by the *in situ* hybridization procedure. Anti-GFP (MBL, 598, 1:500) was used as the primary antibody and anti-rabbit IgG Alexa Fluor 488 (A21206, 1:500, Invitrogen) was used as the secondary antibody. Nuclei were visualized using Hoechst33342 (346-07951, Wako-Fuji film) after performing *in situ* hybridization and immunostaining. Images were captured using an Olympus BX61 fluorescence microscope.

### Measurement of signals from sections

The measurement of gene expression domains was made on binarized images (Fig. S2). We first rotated each image to make the A-P axis horizontal, then measured the A-P length of the blastema mesenchymal region. The image was binarized using the threshold tool in ImageJ (Fiji). As the *in situ* hybridization signal intensity varied among samples, we set a threshold value for each sample. Automatic determination of the threshold was challenging, so the threshold values were manually set by visual inspection. We then removed auto-fluorescent signals, mainly derived from blood cells, manually. Finally, the coordinates of the anterior and the posterior ends of the binarized signals were obtained. As gene expression domains were varied at the proximodistal level, we selected two or three sections from an identical blastema, on which *Shh* and *Fgf8* expression could be confirmed, and calculated their average. To maintain fairness, all measurements were performed under the same exposure time and magnification.

### Nonlinear regression

Regression to the allometric equation was performed using the Mathematica (ver. 12.1.1.0) FindFit function (in Figs 1A,B,F,G, 2J-M, 3J-K, 5O,P). The bold lines represent the regression results for the observed data, whereas the thin lines represent the regression results for the bootstrapped sample data. In Fig. 4M,N, the regression was performed using Python's GPy library for the Gaussian process.

### Electroporation

The animals were anesthetized as described above and the DNA construct was injected directly into the blastema. Immediately after injection, electric pulses were applied (20 V, 50 ms pulse length, 950 ms interval, 10 times) (NEPA21, Nepa gene). The injected DNA constructs were as follows: pCS2-AcGFP and pCS2-*Shh*-p2a-AcGFP. *Shh*-p2a-AcGFP were created as artificial synthetic genes and subcloned into the pCS2 vector. All plasmids were purified with a Genopure Maxi kit (Roche). The blastema samples were fixed 5 days after electroporation.

### A mathematical model for SHH diffusion and *Ptch1* expression

In Fig. 3L-N, we solved the following partial differential equation for diffusion and linear degradation of SHH to examine how the spatial profile of SHH depends on the blastema size and the source size *Shh*(+):

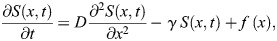


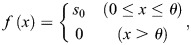
where *S*(*x*, *t*) is the SHH concentration at a position (along the anteroposterior axis) *x* and time *t*. *D* and *γ* are the diffusion constant and degradation rate, respectively. *θ* is the size of the *Shh* expression domain [*Shh*(+)], which depends on the blastema size *L* with an allometric relationship θ = 2.1L^0.69^ (Fig. 2J). *s*_0_ is the secretion rate of SHH from *Shh*(+). The neumann boundary condition (∂*u*/∂*x*=0) was applied at both the posterior end (*x*=0) and anterior end (*x*=*L*) of the blastema. Assuming that the SHH concentration varies smoothly at *x*=*θ*, the solution to this differential equation at the steady state is as follows:

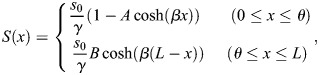
where *A* = sinh(*β*(*L*−*θ*))/sinh(*βL*), *B* = sinh(*βθ*)/sinh(*βL*) and 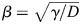. Then, considering that *Ptch1* expression is activated by SHH at each location, its spatial profile is given by the Hill equation as follows:

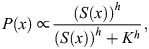
where *K* is a constant giving the half-maximal response. In Fig. 3M,N, the following parameter values were used: *s*_0_/*γ*=1, *β*^−1^=0.3(mm), *L*=0.75-2.5 (mm), *K*=0.25 and *h*=4.

### The spatial profile graph of Hoechst and BrdU signal density

After performing immunofluorescent and Hoechst staining on identical sections, captured images were transferred to Fiji software for analysis. The signals on the epithelial region were deleted manually, then the Hoechst or BrdU signals were selected using the color threshold tool. After being transformed into a 16-bit image, the image was binarized using the threshold tool. The intensity of the selected BrdU or Hoechst signals were projected along the *x*-axis and the values were obtained by the plot profile tool. The values were normalized by the blastema width along the *y*-axis. Finally, the values were normalized by the median and plotted in a graph. The gray and black lines represent the simple moving average of 50 intervals of BrdU or Hoechst signal intensity.

### Cell culture and protein addition

Harvested blastemas were removed from epithelial tissues in 1% EDTA/PBS using forceps. Only mesenchyme tissues were used for the cell culture. After washing with PBS, the tissues were broken into smaller pieces. Obtained mesenchyme cells were suspended in culture medium [50% water, 40% GlutaMax-DMEM, 10% FCS and 0.01 M HEPES (pH. 7.5) and 300 mg/ml Gentamycin]. FGF8 (R&D Systems, 423-F8) and/or SHH (R&D Systems, 461-SH) proteins were added to the culture medium at a concentration of 0.1 µg/ml. The culture medium was refreshed every day.

### qRT-PCR analysis

RNA preparation for RT-PCR was performed using TriPure Isolation Reagent (Roche). Reverse transcription was performed using PrimeScript II Reverse Transcriptase (Takara). The quantitative RT-PCR analysis was performed by StepOneTM (ThermoFisher) and KAPA SYBR FAST qPCR Master Mix (Kapa Biosystems). Primers are described below: *EF1α* forward, AACATCGTGGTCATCGGCCAT; *EF1α* reverse, GGAGGTGCCAGTGATCATGTT; *Pcna* forward, ATGTTTGAGGCTCGCCTGGT; *Pcna* reverse, GCAGCGGTACGTATCGAAGC.

### Whole-mount Alcian Blue staining

Samples were fixed with 10%-Formaldehyde Neutral Buffer Solution (37152-51, Nakalai Tesque) for 1 day. Samples were then stained with Alcian Blue solution (Wako, pH 2.0) and dissolved in 70% ethanol/1% HCl for 1 day after replacing the 10% formalin solution with 70% ethanol. The samples were washed with 70% ethanol, then placed in 100% ethanol.

### 3D reconstruction

Two or three series of consecutive blastema sections underwent *in situ* hybridization, BrdU and GFP immunofluorescence, and nuclei visualization by Hoechst 33342. Images were captured using an Olympus BX61 fluorescence microscope and were reconstructed through volume rendering using Amira Software (version 6.4.0, Thermo Fisher Scientific; http://www.fei.com/software/amira-3d-for-life-sciences/) running on an iMacPro (CPU, 2.3 GHz Intel Xeon W; DRAM, 128GB 2666 MHz DDR4; and graphics, Radeon Pro Vega 64 16368 MB). Gene expression domains and GFP signals were extracted using the segmentation tool on Amira. BrdU signals were extracted using the segmentation tool in Amira, and merged signals were isolated using the watershed tool in ImageJ (Fiji). BrdU coordinate information was obtained using the connected components tool in Amira. Hoechst signals were extracted using the segmentation tool, and a heatmap was created using the DistanceMap tool in Amira.

## Supplementary Material



10.1242/develop.202697_sup1 Supplementary information
