# Supplementary material for: Allometry in limb regeneration and scale-invariant patterning as the basis of normal morphogenesis from different sizes of blastemas
Source: Development. 2024 Nov 8;151(21):dev202697. doi: 10.1242/dev.202697 (PMC11574362; doi:10.1242/dev.202697)
Supplement: Supplementary information [file develop-151-202697-s1.pdf]

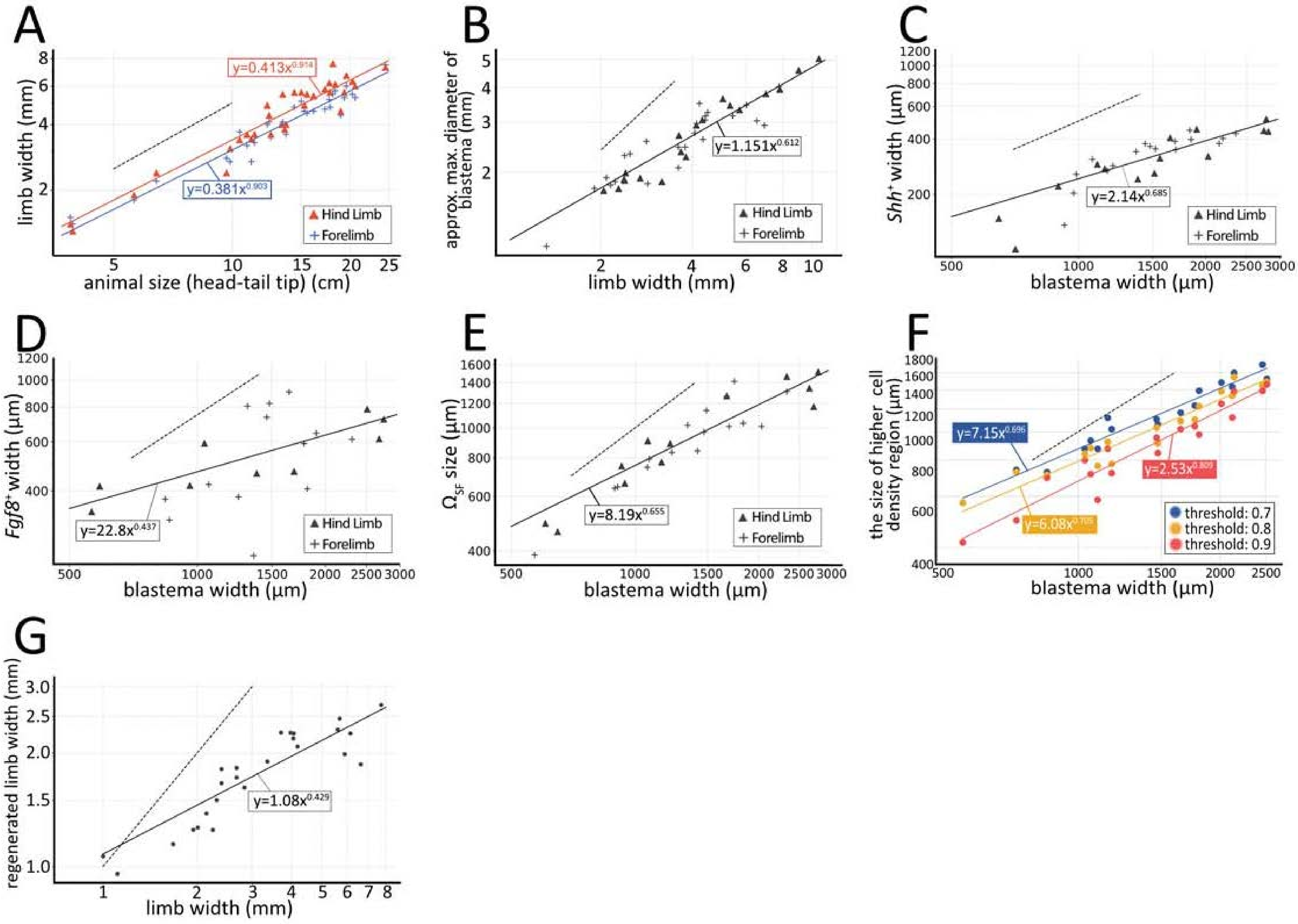

Fig. S1. The main figure scatter plot represented as double logarithmic graphs

To visualize the exponent values as slopes, the scatter plots from the main figures are here transformed into double logarithmic graphs. The solid lines in each graph show the regression curve. For reference, the dotted lines show when the exponent is equal to 1. The value of coefficient a on the dotted line has been adjusted to make it easier to compare with the regression curve. (**A**) Limb size scales isometrically (the solid and the dotted lines are nearly parallel) with animal size. (**B**) The allometric relationship between blastema and stump-limb size. (**C and D**) The relationship between the width of *Shh*(+) or *Fgf8*(+) and the width of the blastema mesenchyme (Fig.2 **J** and **L**). The *Shh*(+) scales nearly isometrically (the solid and the dotted lines are roughly parallel) and the *Fgf8*(+) scales allometrically. (**E**) Ω_{SF} size scales allometrically with blastema size (Fig. 3**J**). (**F**) The relationship between the sizes of regions with cell densities above certain thresholds and blastema sizes (Fig. 4**P**). (**G**) The allometric relationship between regenerated limb sizes and stump limb sizes (Fig. 5**O**).

Development • Supplementary information

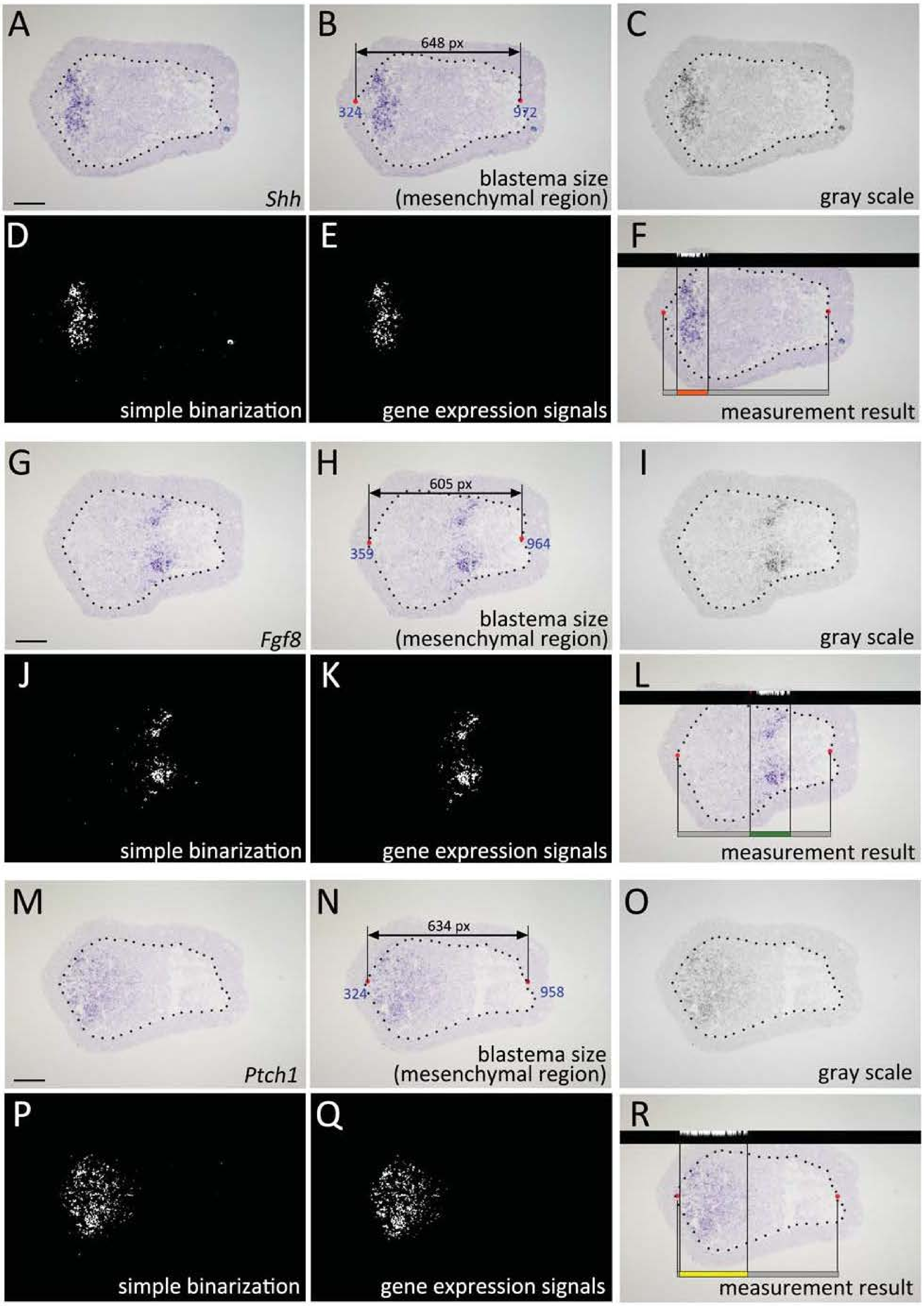

Development • Supplementary information

Fig. S2. The method for measuring gene expression signals

The measurement method for *Shh* (**A–F**), *Fgf8* (**G–L**), and *Ptch1* (**M–R**) expression signals. (**A–C, G–I**, and M–O) The images were rotated to make the A P axis horizontal, the A−P length of the blastema mesenchymal region was measured and, the images were converted to black-and-white. (**D–F**, **J–K**, and **P–R**) The images were binarized and non-specific signals were manually removed. The coordinates of the anterior and the posterior ends of the binarized signals were then obtained.

Development • Supplementary information

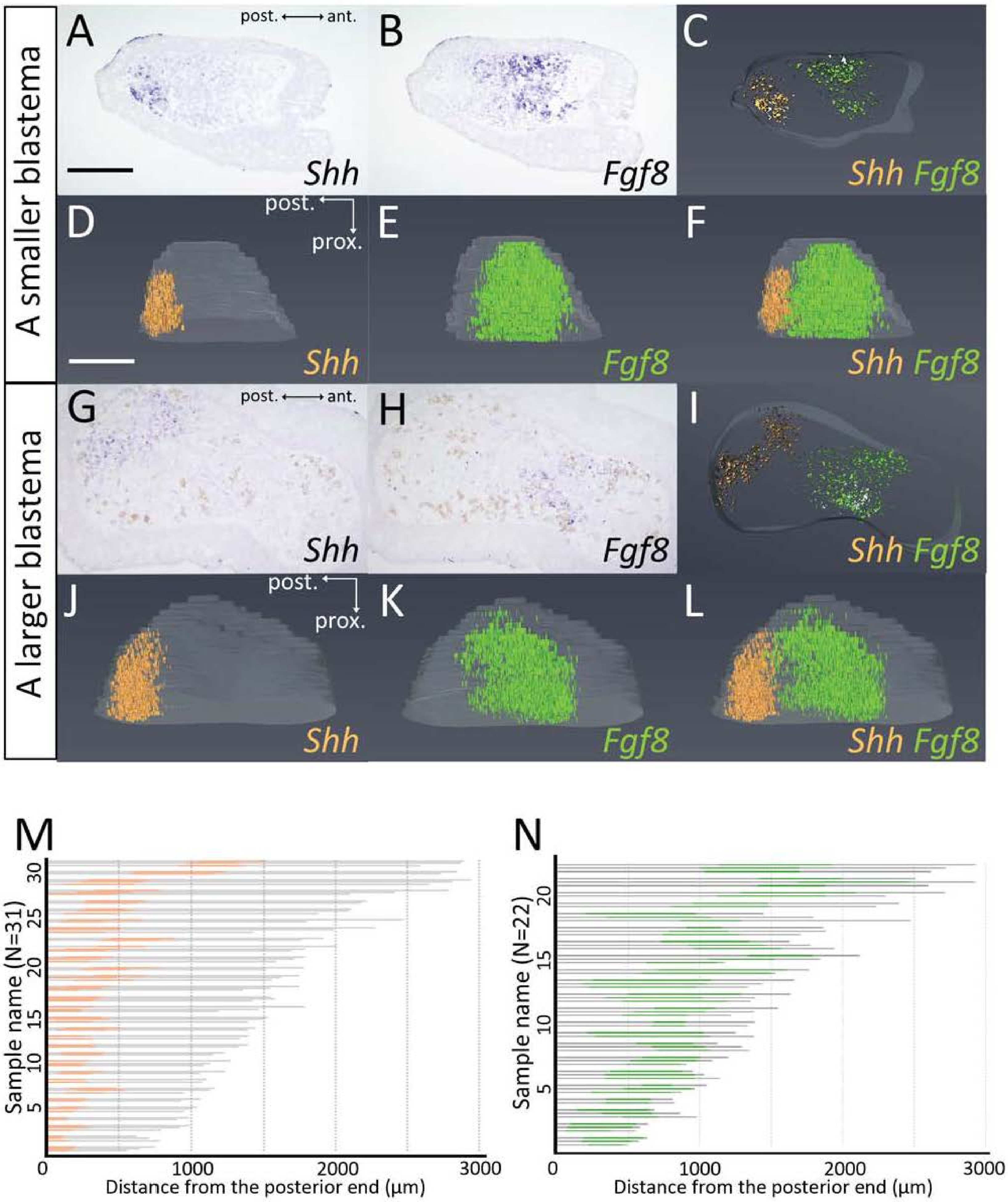

Fig. S3. Reconstituted *Shh* and *Fgf8* expression patterns in different sizes of blastema and one-dimensionalized *Shh* and *Fgf8* expression

(**A–L**) A series of sections were made throughout the blastema and reconstituted into three-dimensional images for visualization of the spatial distribution of the *Shh*(+) and the *Fgf8*(+). (**A–F**) A smaller sized blast ema. (**G–L**) a larger sized blastema. (**A, B, G, H**) Selected sections showing typical *Shh* and *Fgf8* expression patterns in the blastema. (**C–F**) and (**I–L**) are the reconstituted images showing *Shh*(+) cells (orange), *Fgf8*(+) cells (light green), and the border of the blastema mesenchyme (gray). (**C, L**) The proximal view of the proximal six sections. Scale bar in (**A**) and (**D**): 400 µm. (**M, N**) Raw data for the *Shh*(+) (**M**) and *Fgf8*(+) (**N**) widths. The measurements were performed on 2 to 3 sections derived from identical blastemas. Lines indicate the results from all measurements of 2 to 3 sections. Lines were bundled at every blastema and displayed. (n = 31 (*Shh*), n = 22 (*Fgf8*)).

Development • Supplementary information

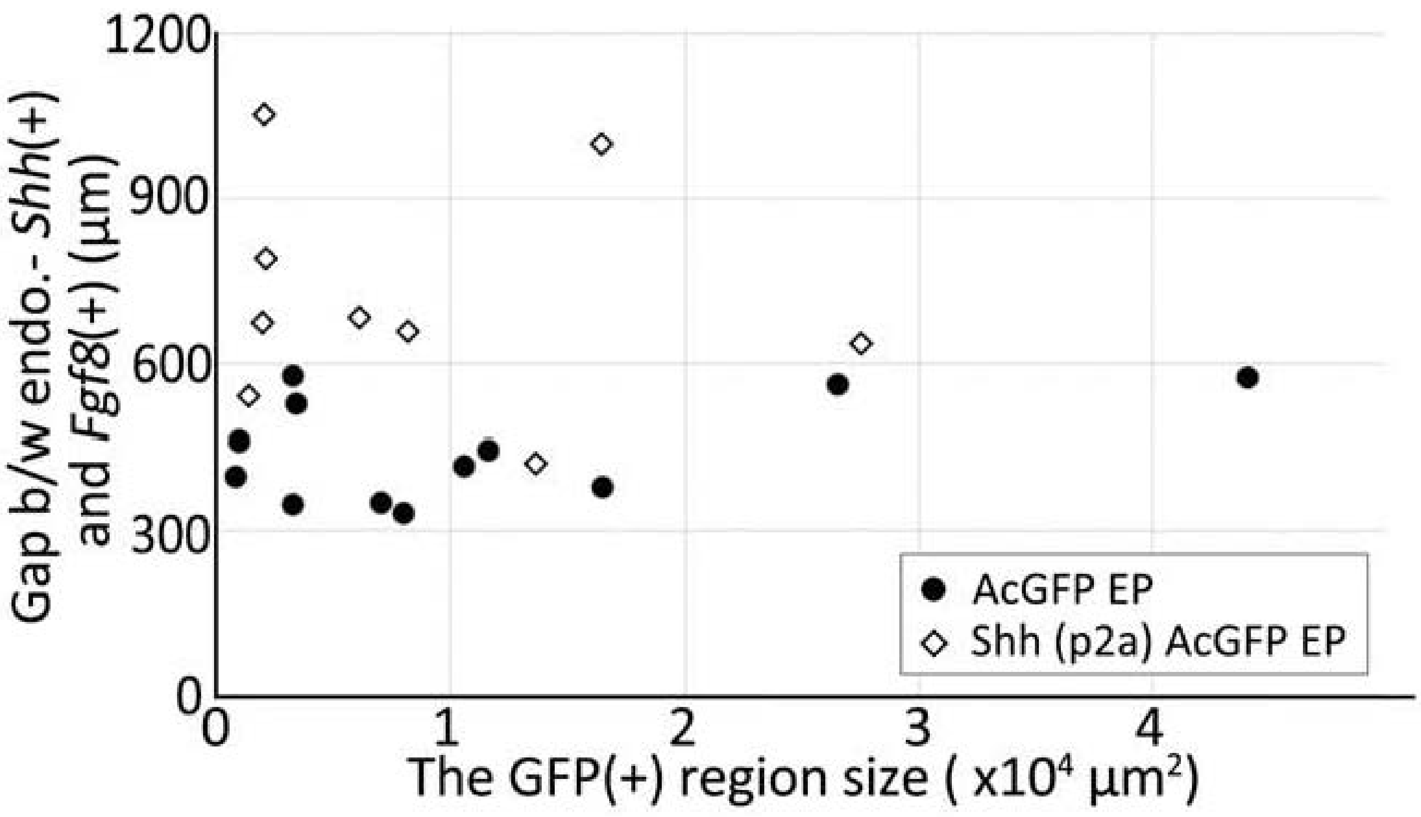

Fig. S4. The relationship between the total area of the GFP(+) regions and the gap size between the endogenous *Shh*(+) and *Fgf8*(+)

The vectors, pCS2–AcGFP or pCS2–Shh–p2a–AcGFP were electroporated into the blastema mesenchyme. The scatter plot shows the relationship between the total area of GFP(+) and the size of the endogenous *Shh*(+) and *Fgf8*(+) gap. The correlation coefficients R were 0.46 for pCS2–AcGFP EP (n = 12) and -0.13 for pCS2–Shh–p2a– AcGFP EP (n = 8), respectively.

Development • Supplementary information

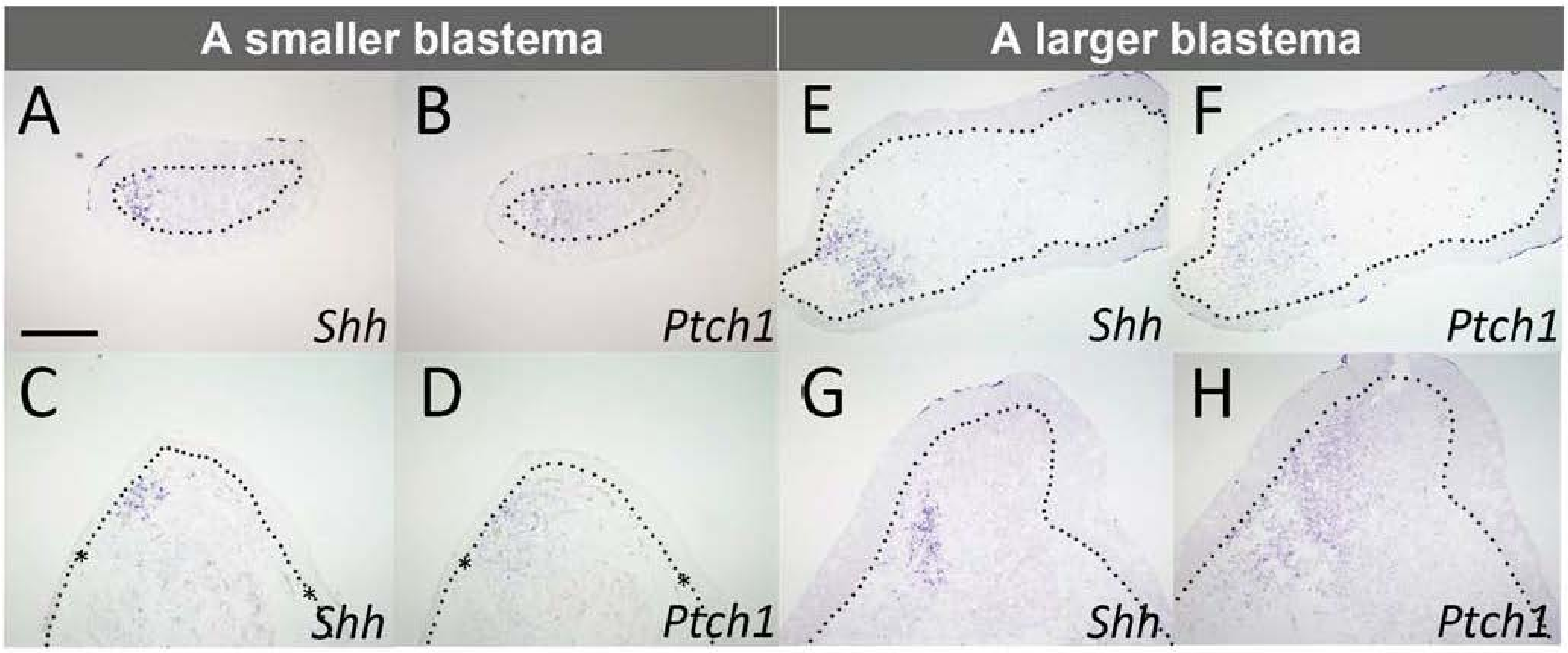

Fig. S5. Expression of *Ptch1* in the posterior region of the blastema mesenchyme, covering the *Shh*(+)
(**A–H**) *Shh* or *Ptch1* expression was visualized by *in situ* hybridization in a smaller blastema (**A–D**) and a larger blastema (**E–H**). Dotted lines indicate the border of the blastema epithelium. Asterisks in (**C**), (**D**), (**G**), and (**H**) denote the discontinuity in dermal collagen, indicating the amputation site. Scale bar in (**A**): 400µm. (**A–H**) are the same magnification.

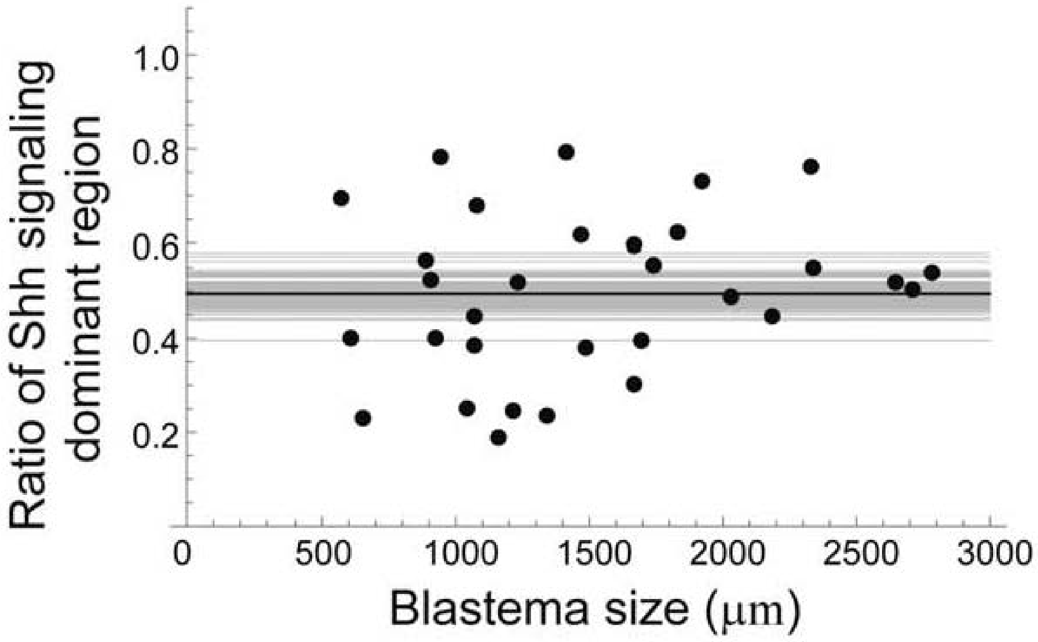

Fig. S6. The relative sizes of the Shh signaling dominant regions within the Ω_{SF}
The scatter plot shows the relationship between the relative sizes of the Shh signaling dominant regions within the Ω_{SF} and blastema sizes (n = 31). The p-value for a correlation test (null hypothesis: no correlation) was 0.2, and the null hypothesis was not rejected.

Development • Supplementary information

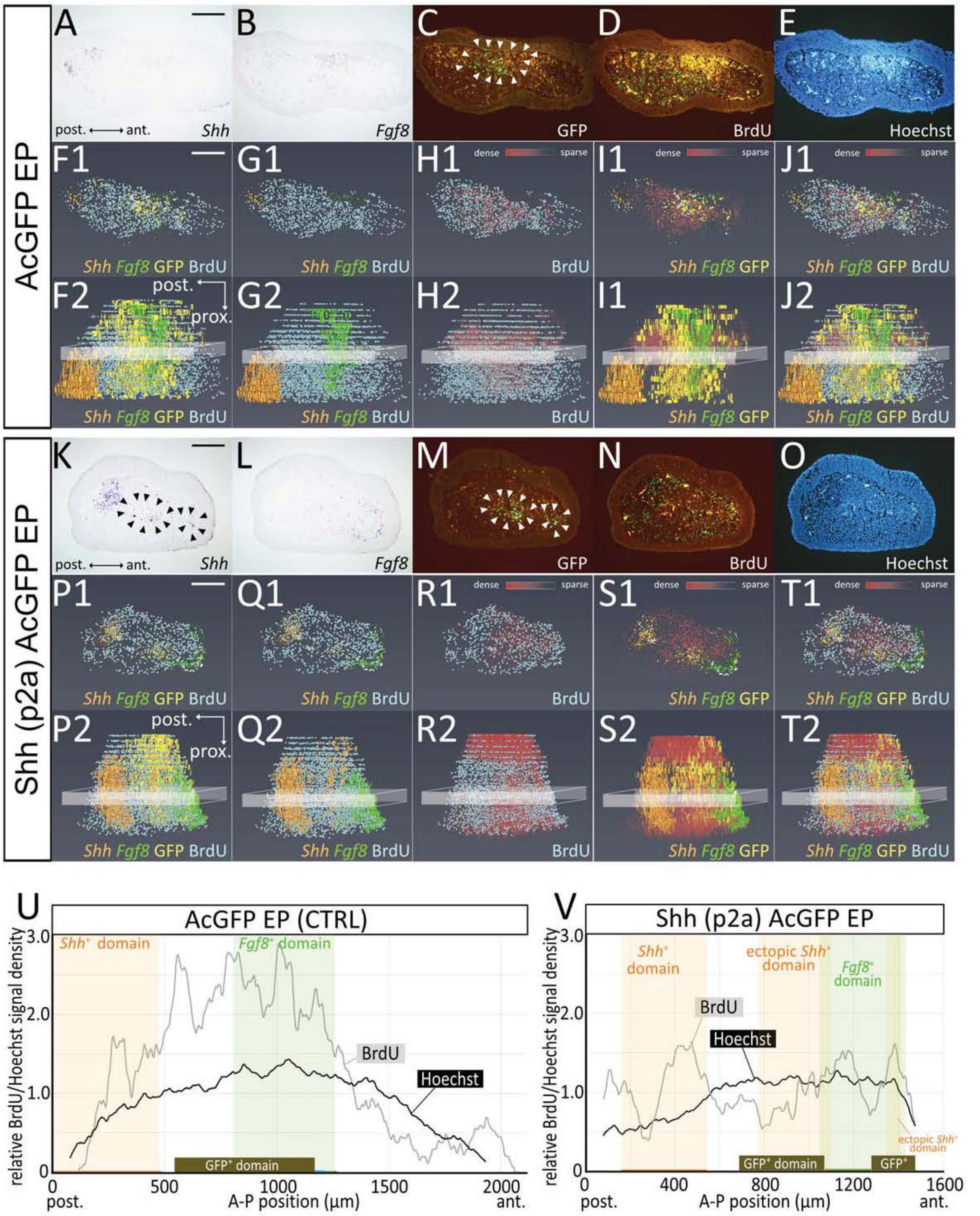

Fig. S7. Artificially induced anterior shift of Ω_{SF} position in blastema resulting in anteriorly shifted cell density and cell proliferation activity

(**A–T**) The vectors, pCS2–AcGFP (**A–J**) or pCS2–Shh–p2a–AcGFP (**K–T**), were electroporated into the blastema mesenchyme, and the effects of the ectopic gene expression on the cell density and proliferation activity were verified. (**F–J**, **P–T**) Reconstituted three-dimensional images. (**F1–J1, P1–T1**) Proximal views of the indicated areas in (**F2–J2**), (**P2–T2**). Nine sections were extracted from the blastema. *Shh*: orange, *Fgf8*: light green, GFP: yellow, BrdU: light blue, cell density: heatmap. Scale bars in (**A**) and (**K**): 400 μm, in (**F1**) and (**P1**): approximately 400 μm. (**U, V**) Spatial profiles of cell density (Hoechst, black) and proliferation activity (BrdU, gray) quantified from the image data in (**A–J**) and (**K–T**).

Development • Supplementary information

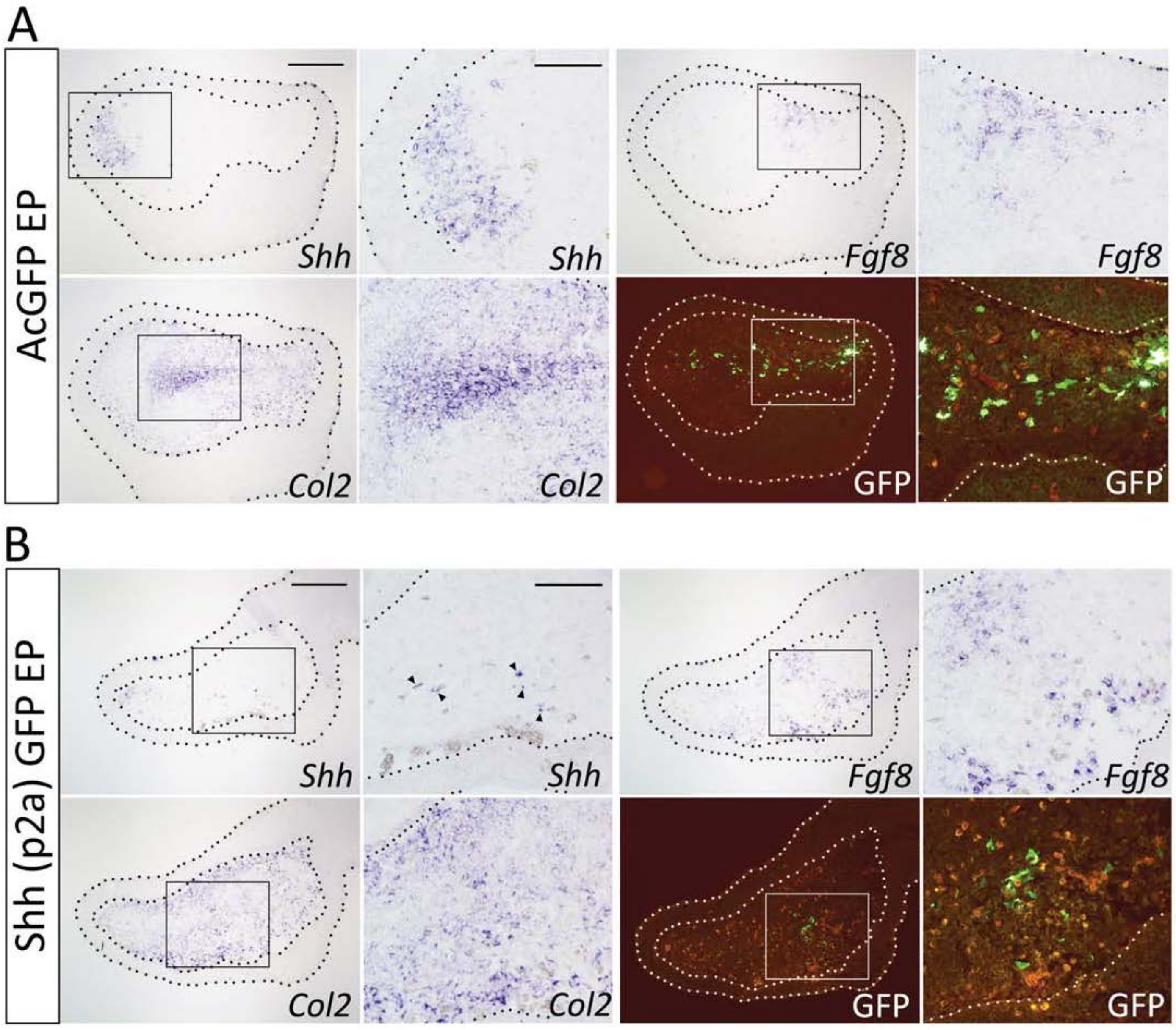

Fig. S8. The delay of the digit differentiation due to the ectopic induction of *Shh* expression (**A**—**H**) The vectors, pCS2−AcGFP (**A−D**) or pCS2-Shh−p 2a−AcGFP (**E−H**) were electroporated into the blastema mesenchyme. High magnification images of the squared region in **A−H** are shown in **A'−H'**. The construct was electroporated at a slightly later stage than in the case of Fig. 3**C** and Fig. S7. When the pCS2−AcGFP vector was introduced, the *Col2*–expressing cells showed an aggregation in most samples (n = 5/6) (**A−D**). However, when the pCS2−Shh−p 2a−AcGFP was introduced, the concentrated expression pattern of *Col2* was not observed; instead, its expression signals appeared sparsely throughout the entire mesenchymal region (n = 4/5) (**E−H**). Arrowheads in (**E'**) show ectopic *Shh* expression signals. The dotted curves indicate the boundary of the blastema epithelium. Scale bars in (**A**) and (**E**): 400μm, in (**A'**) and (**E'**): 200μm.

Development • Supplementary information

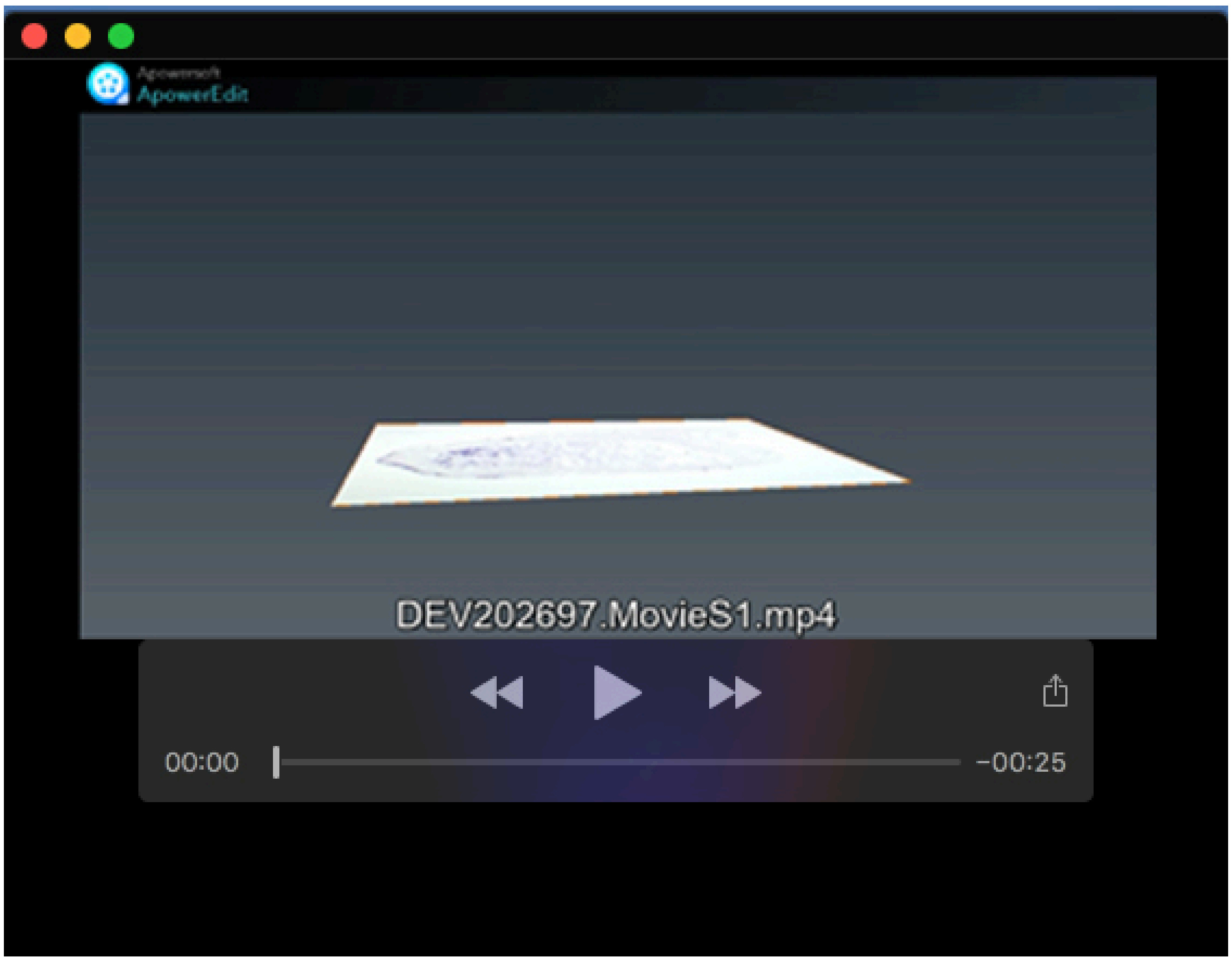

Movie 1. The spatial expression pattern of *Shh* (orange) and *Fgf8* (light green) in a smaller blastema. The image is reconstituted from the sections, in which gene expression was visualized by *in situ* hybridization. Light gray shows the boundary between the mesenchyme and the epithelium. Snapshots from this movie are used in Fig. S2**C–F**.

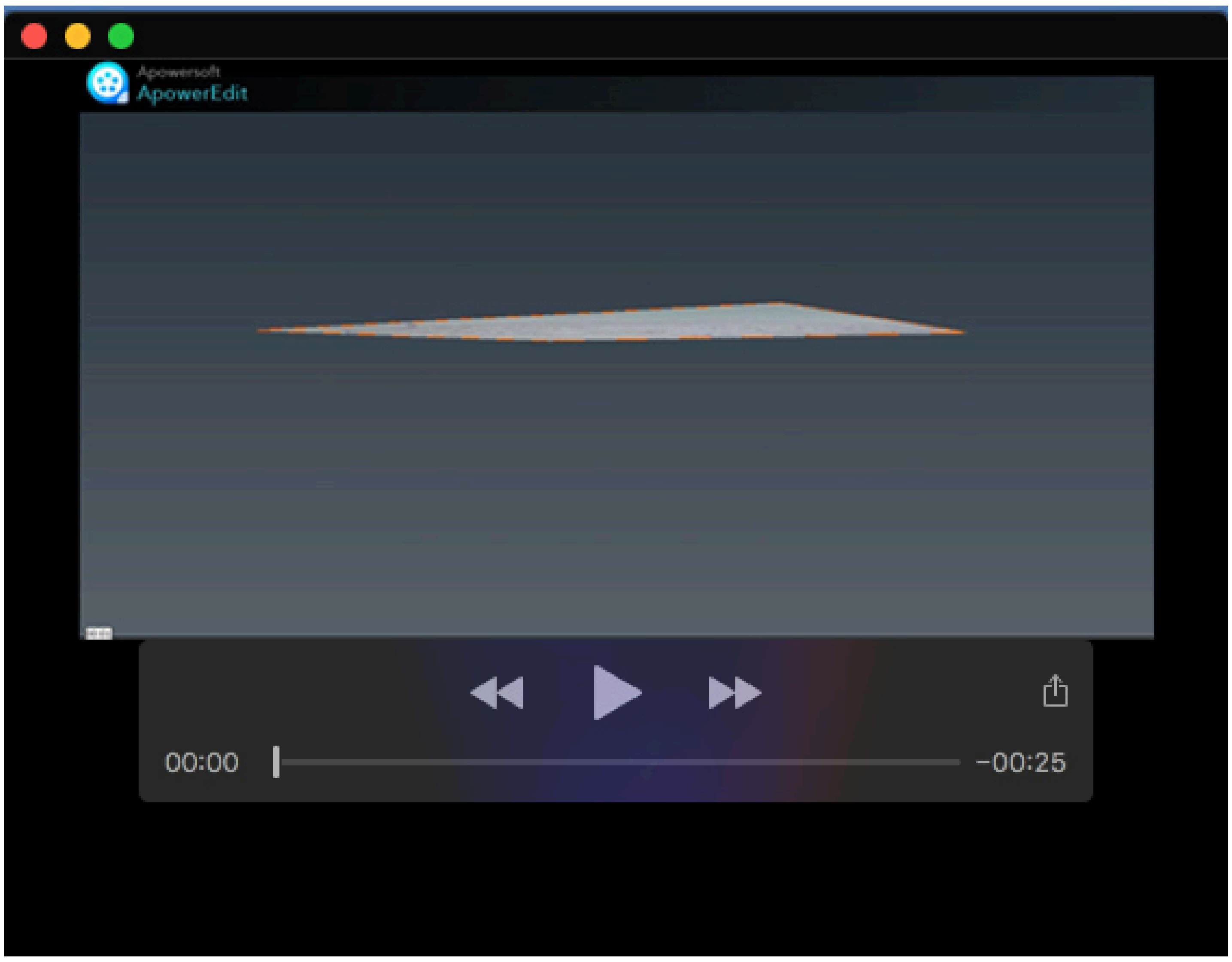

Movie 2. The spatial expression pattern of *Shh* (orange) and *Fgf8* (light green) in a larger blastema. The image is reconstituted from the sections, in which gene expression was visualized by *in situ* hybridization. Light gray shows the boundary between the mesenchyme and the epithelium. Snapshots from this movie are used in Fig. S2**I–L**.

Development • Supplementary information

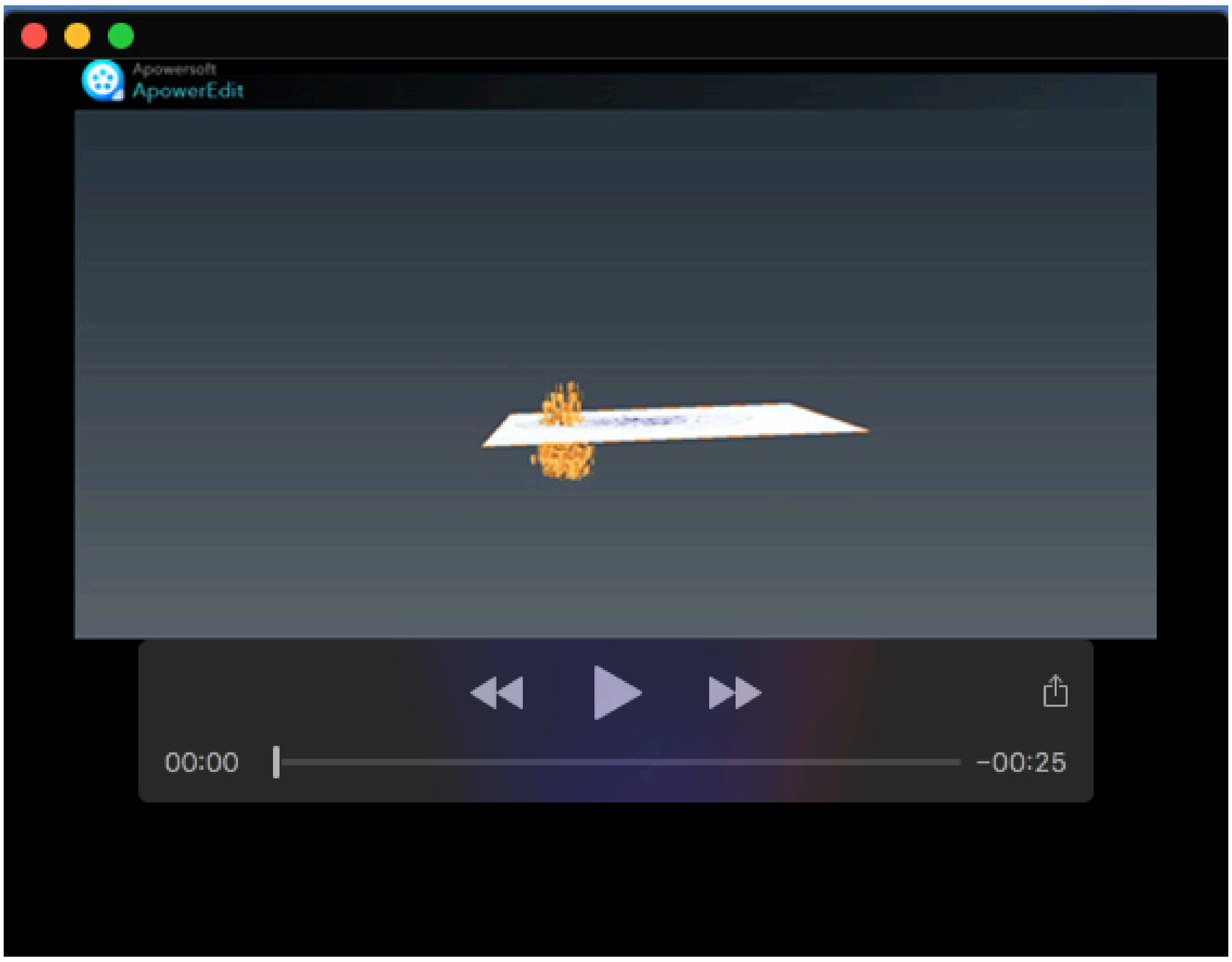

Movie 3. The spatial expression pattern of *Shh* (orange) and *Fgf8* (light green) in a smaller blastema. The distribution of BrdU signals (light blue) and the cell density heatmap (deep brown to deep gray) are also visualized.

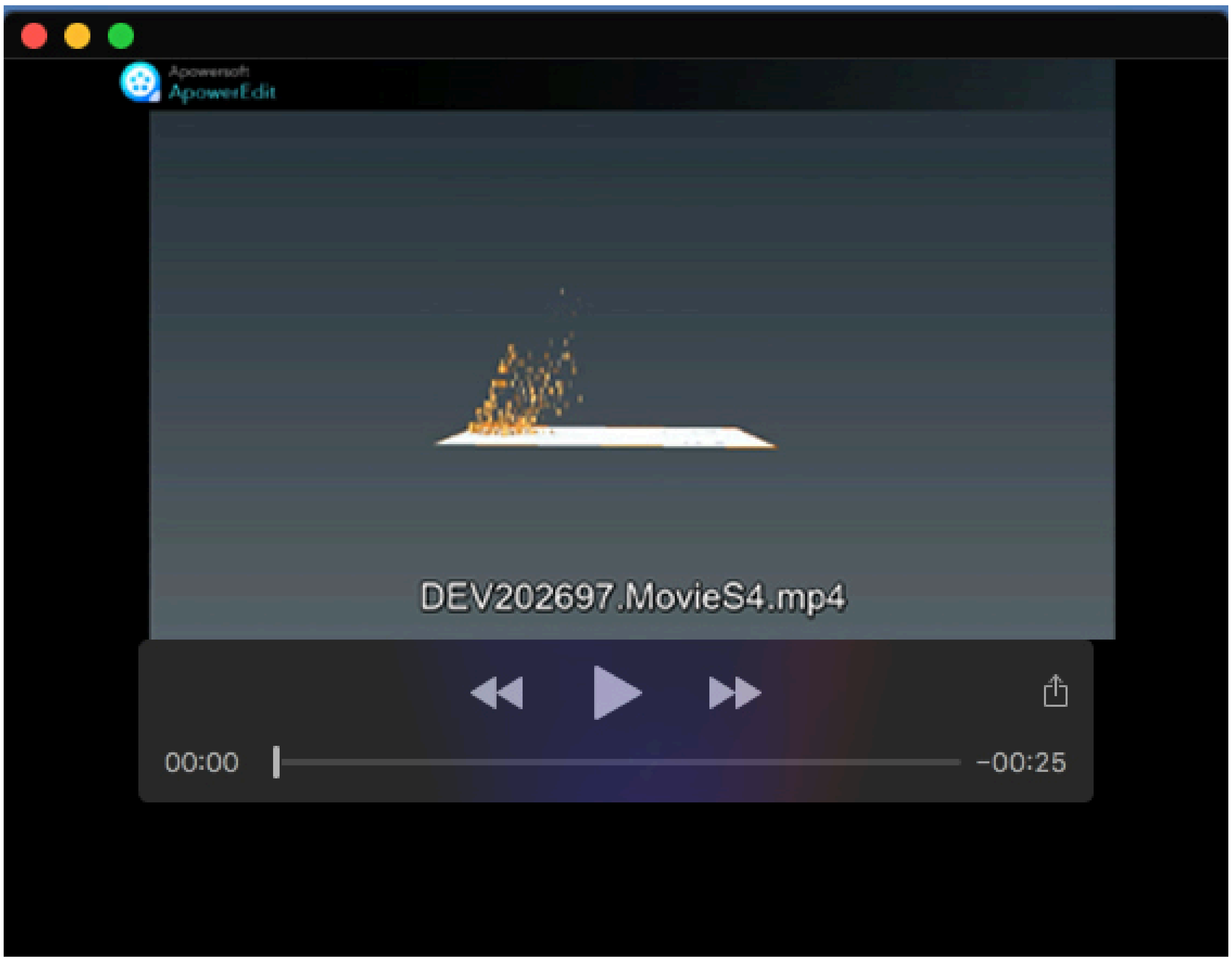

Movie 4. The spatial expression pattern of *Shh* (orange) and *Fgf8* (light green) in a larger blastema. The distribution of BrdU signals (light blue) and the cell density heatmap (deep brown to deep gray) are also visualized.

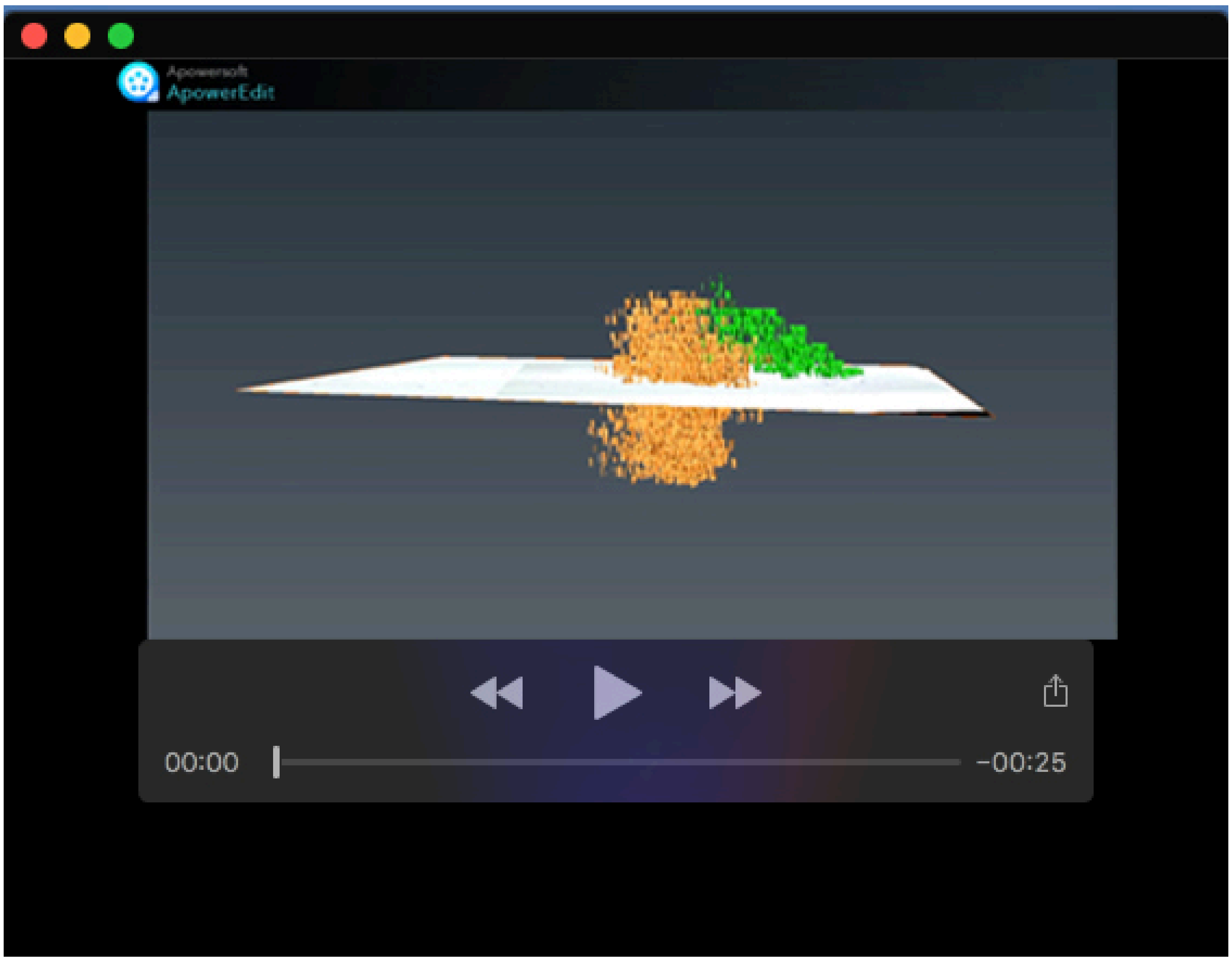

Movie 5. The spatial expression pattern of *Shh* (orange) and*Fgf8* (light green) in an anteriorly shifted medium-size blastema. The distribution of BrdU signals (light blue) and the cell density heatmap (deep brown to deep gray) are also visualized.

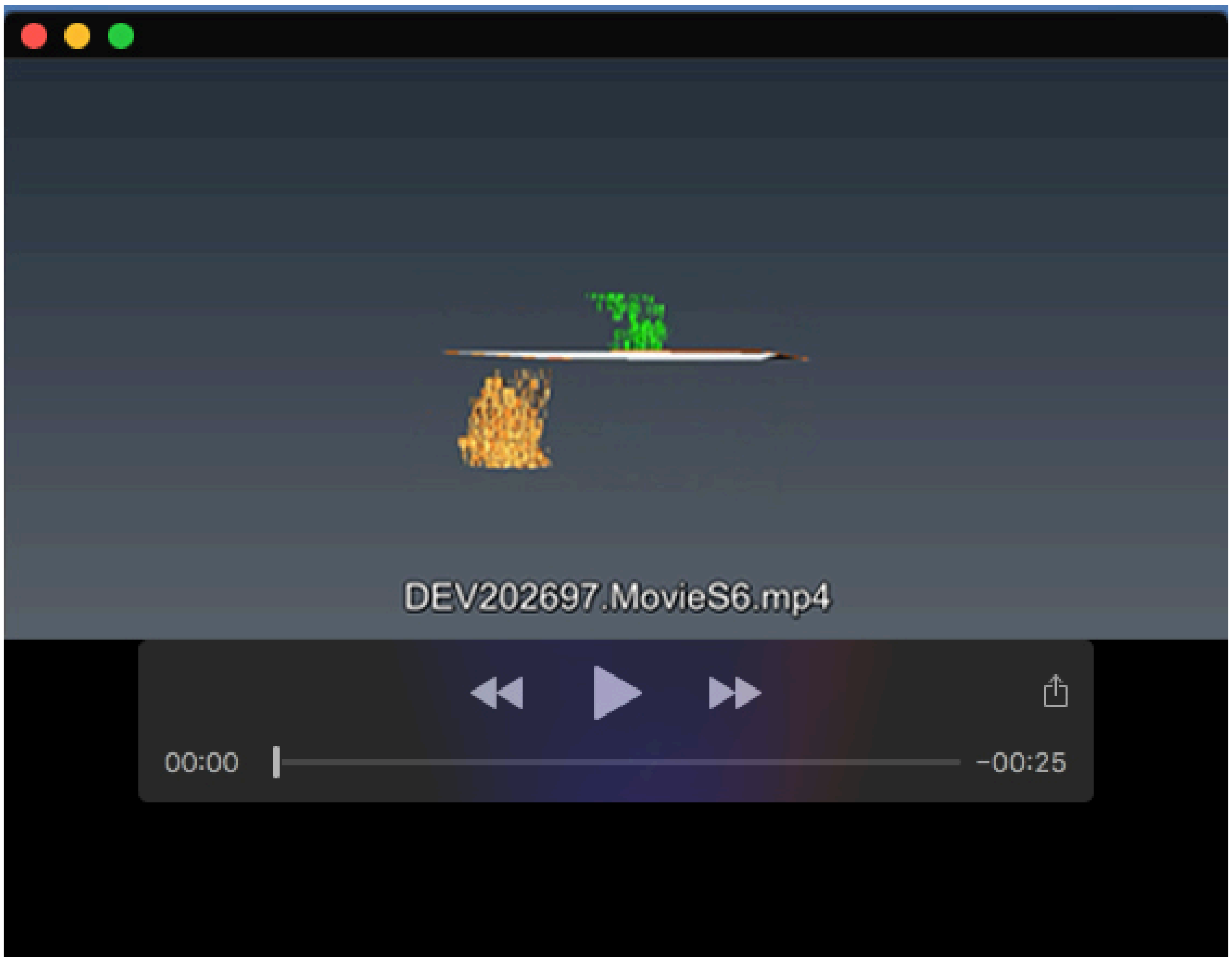

Movie 6. The spatial pattern of *Shh* (orange), *Fgf8* (light green), and GFP (yellow) in a blastema electroporated by the pCS2–AcGFP vector. The distribution of BrdU signals (light blue) and the cell density heatmap (deep brown to deep gray) are also visualized. Snapshots from this movie are used in Fig. S4**F–J**.

Development • Supplementary information

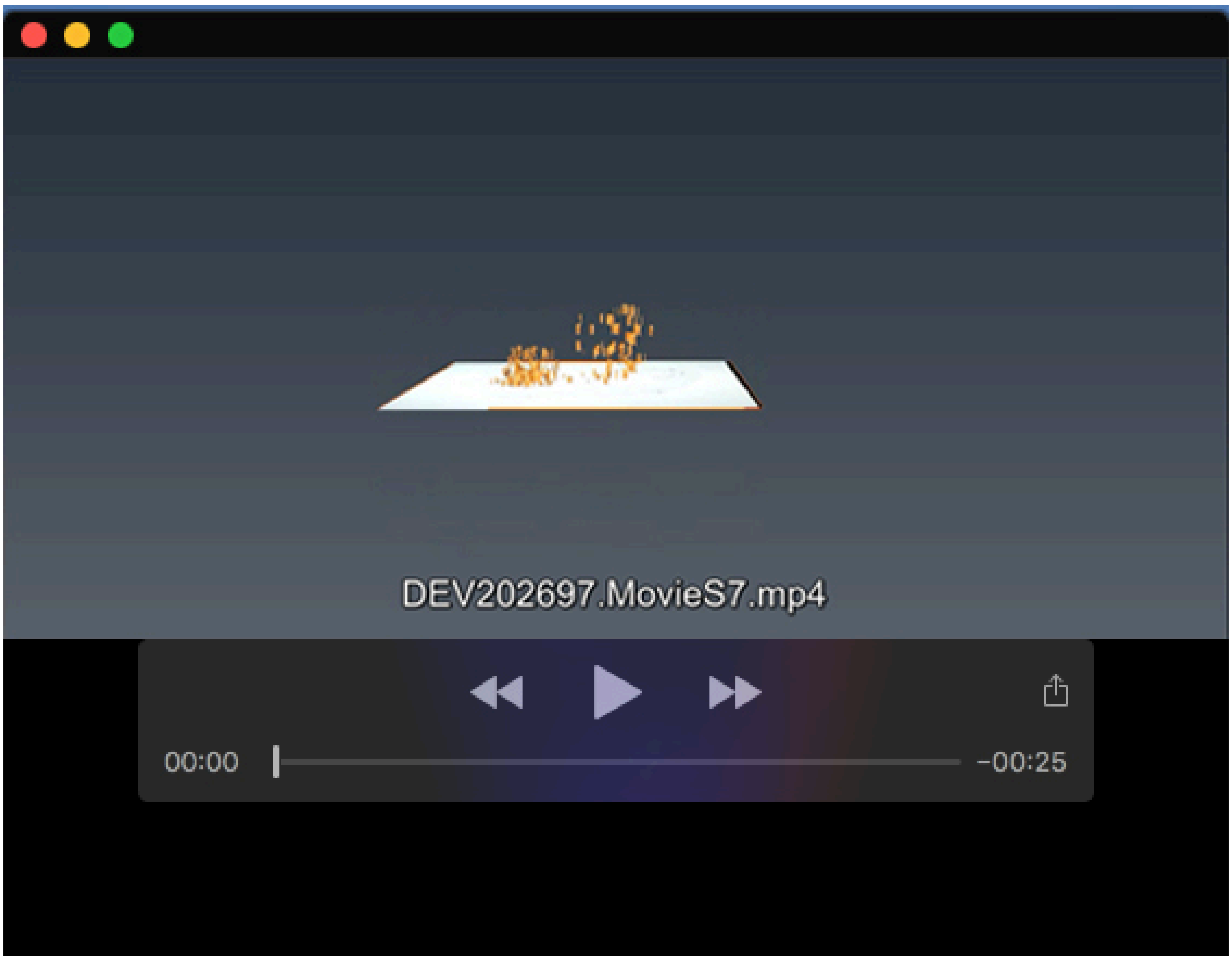

Movie 7. The spatial pattern of *Shh* (orange), *Fgf8* (light green), and GFP (yellow) in a blastema electroporated by the pCS2–Shh–p2a–AcGFP vector. The distribution of BrdU signals (light blue) and the cell density heatmap (deep brown to deep gray) are also visualized. Snapshots from this movie are used in Fig. S4**P–T**.
