# Supplementary material for: Allometry in limb regeneration and scale-invariant patterning as the basis of normal morphogenesis from different sizes of blastemas
Source: Development. 2024 Nov 8;151(21):dev202697. doi: 10.1242/dev.202697 (PMC11574362; doi:10.1242/dev.202697)
Supplement: Supplementary file 1 [file develop-151-202697_review_history.pdf]

Allometry in limb regeneration and scale-invariant patterning as a basis of normal morphogenesis from different sizes of blastemas

Saya Furukawa, Sakiya Yamamoto, Ayaka Ohashi, Yoshihiro Morishita and Akira Satoh
DOI: 10.1242/dev.202697

Editor: Kenneth Poss

Review timeline

Original submission:	10 January 2024
Editorial decision:	20 March 2024
First revision received:	3 July 2024
Editorial decision:	22 July 2024
Second revision received:	9 September 2024
Accepted:	23 September 2024

Original submission

First decision letter

MS ID#: DEVELOP/2024/202697

MS TITLE: Allometry in limb regeneration and scale-invariant patterning as a basis of normal morphogenesis from different sizes of blastemas

AUTHORS: Saya Furukawa, Sakiya Yamamoto, Ayaka Ohashi, Yoshihiro Morishita, and Akira Satoh

I have now received all the referees' reports on the above manuscript, and have reached a decision. The referees' comments are appended below, or you can access them online: please go to BenchPress and click on the 'Manuscripts with Decisions' queue in the Author Area.

As you will see, the referees express considerable interest in the question your work pursues and in your findings, but have some significant criticisms and recommend a substantial revision of your manuscript before we can consider publication. If you are able to revise the manuscript along the lines suggested, which may involve further experiments, I will be happy to receive a revised version of the manuscript. Your revised paper will be re-reviewed by one or more of the original referees, and acceptance of your manuscript will depend on your addressing satisfactorily the reviewers' major concerns. Please also note that Development will normally permit only one round of major revision. If it would be helpful, you are welcome to contact us to discuss your revision in greater detail. Please send us a point-by-point response indicating your plans for addressing the referees' comments, and we will look over this and provide further guidance.

Please attend to all of the reviewers' comments and ensure that you clearly highlight all changes made in the revised manuscript. Please avoid using 'Tracked changes' in Word files as these are lost in PDF conversion. I should be grateful if you would also provide a point-by-point response detailing how you have dealt with the points raised by the reviewers in the 'Response to Reviewers' box. If you do not agree with any of their criticisms or suggestions please explain clearly why this is so.

Reviewer 1

Advance summary and potential significance to field

© 2024. Published by The Company of Biologists under the terms of the Creative Commons Attribution License (https://creativecommons.org/licenses/by/4.0/).

Furukawa et al seek to understand the nature of the scaling laws that underlie the growth of limb blastema to achieve normal morphogenesis from different size start points. This is an important question which has seldom been tackled at the quantitative level and I commend the authors for doing so.

In a nutshell, the authors investigate the relationship between various parameters (blastema, stump or body size, Shh, Ptch1, FGF8 expression areas as well as relative A-P based sub-areas) and uncover important principles underlying blastema growth: that scaling is allometric, that the ratio of shh-fgf8 signaling-dominant regions is invariable, that Shh expression scales with blastema size, that maximum cell density & proliferation scale together with the Shh-Fgf8 signaling-dominant region, and provide a mathematical description for the growth scaling from digit formation to regenerating limb prior to secondary growth.

Comments for the author

Furukawa et al seek to understand the nature of the scaling laws that underlie the growth of limb blastema to achieve normal morphogenesis from different size start points. This is an important question which has seldom been tackled at the quantitative level and I commend the authors for doing so.

In a nutshell, the authors investigate the relationship between various parameters (blastema, stump or body size, Shh, Ptch1, FGF8 expression areas as well as relative A-P based sub-areas) and uncover important principles underlying blastema growth: that scaling is allometric, that the ratio of shh-fgf8 signaling-dominant regions is invariable, that Shh expression scales with blastema size, that maximum cell density & proliferation scale together with the Shh-Fgf8 signaling-dominant region, and provide a mathematical description for the growth scaling from digit formation to regenerating limb prior to secondary growth.

I find this work is highly relevant to the audience of Development, it is original, overall clearly written and discussed, and brings forward further research questions. As such I would encourage its publication.
Nevertheless, there are a few suggestions and comments to consider ahead of this:

-The study addresses relationships which are applicable to blastema size yet all data derives from lower arm amputations. We know that there is a difference in blastema growth between upper and lower arm amputations. It appears from the text that the authors imply that the scaling laws would apply to upper arm blastemas as well, but it would be good to provide evidence that this is the case (at least regarding shh-fgf8 scaling), or else discuss it.

-are the results from ectopic Shh expression (Fig 3A-C) corrected by electroporation efficiency?

-Suppl Fig 4 U vs V : there is not only a shift in the proliferation peak but there appears to be an overall decrease or flattening of the proliferation profile overall – please discuss.

-When the authors shift Shh domain by electroporation, and therefore the area of maximums density (as shown in Suppl Fig 4), do they shift also the position of Col2A expression?

-P. 7 line 142 ‘little correlated’ should be changed to ‘it does not correlate well’

-P. 9 line 188 change ‘special’ to ‘spatial’

-Figure legends: check formatting of panels (bold vs normal)

-Fig. 1: consistency of coefficient annotation (A vs B), add scales to D and E

Reviewer 2

Advance summary and potential significance to field

© 2024. Published by The Company of Biologists under the terms of the Creative Commons Attribution License (https://creativecommons.org/licenses/by/4.0/).

Furukawa et al. present here original research investigating the scaling of blastema patterning in axolotl limb regeneration. The question is very intriguing as axolotls can regenerate limbs of different sizes and it is unclear how signalling patterns and growth rates are adjusted for correct morphogenesis. The manuscript takes a descriptive approach, focusing on the describing patterns and size relationships. The authors compare the size of the regenerated limb and the activation of two important signalling pathways, i.e. the anteriorly located Fgf8 and the posteriorly located Shh. The authors amputated limbs in the same relative position in animals of different sizes. Initially, limb regeneration reconstructs a small, but properly patterned, limb. Then, this regenerated limb grows isometrically. The authors focused on the first moment of regeneration, in which the initial small limb is regenerated. The authors show that the size of the blastema scales sublinearly with the size of the amputated limb, so that the regenerated limb is relatively smaller in larger animals. Interestingly, the relative size of the Shh-expressing domain is almost constant, while the relative size of the Fgf8-expressing domain decreases with blastema size. The region in between the Shh- and Fgf8-expressing domains show expression of Patched1 (Ptch1), which is downstream of SHH signalling suggesting that it is reached by the SHH protein. The relative size of the entire Shh-Patched1-Fgf8 region decreases with blastema size, but the ratio of the sizes of the SHH, i.e. Shh-Ptch1, and Fgf8 regions is constant. The authors show that the position of the highly proliferative and high-density region of the blastema is constant within the Shh-Ptch1-Fgf8 domain, as well as the relative position of the second digit specification domain.

I find this manuscript appropriate for the readership of Development. I find that the analysis and description of the methods should be strengthened in some parts of the manuscript. I propose to strengthen the mechanistic insights of this manuscript with an analysis and an experiment.

Comments for the author

Major points

1) Most of the manuscript is based on the quantification of the sizes of the Shh-, Ptch1- and Fgf8-expressing domains by in situ hybridization. However, I find that the Methods describe vaguely how those domains are defined at the time of the analysis. I got the impression that they are manually selected, which can lead to biases. Given the importance of these measurements for the manuscript, I request the authors to design an automatic quantification method to define the sizes of Shh-, Ptch1- and Fgf8-expressing domains in each image.

2) The authors switch between discussing sizes in absolute and relative terms across the manuscript, which I find confusing. I invite the authors to focus on the description in relative terms since it seems to be the most relevant for the results.

3) Fig 3H, I. Here, the authors state that the ratio of the Shh-Ptch1 and Fgf8 domains is constant in limbs of different sizes. This is intuitive from Fig. 3H, but it is not as rigorously or as clearly reported as in other parts of the manuscript. I request the authors to report the result with a similar plot as in Fig. 3K.

4) Fig. 3L-N. The authors propose a simple mathematical model to rationalize the observation that the ratio of the sizes of the Shh-Ptch1 and Fgf8 domains is constant. In essence, the authors say that the size of the region reached by the SHH protein does not scale with blastema size, because SHH diffusion and degradation do not. However, it is unclear how the Fgf8 domain would scale properly, besides from the fact of being downstream of SHH signaling. I suggest the authors to clarify this point.

5) In Fig. 2, the authors show that the experimental over-expression of SHH shifted the position of the Fgf8-expressing domain.

a) I request the authors to correlate the shift in position and change in size of the Fgf8 domain with the level of Shh-2a-GFP over-expression. I expect this measurement to help clarify the quantitative relationship between Shh and Fgf8 expression.

© 2024. Published by The Company of Biologists under the terms of the Creative Commons Attribution License (https://creativecommons.org/licenses/by/4.0/).

b) If technically possible, I request the authors to assess the relative positions of the cell proliferation and high-density regions, as well as that of second digit formation, following Shh-2a-GFP over-expression. Do they change? Are results compatible with the model proposed by the authors?

6) I find that the presentation of the data of Fig. 4M-N can be confusing as we cannot assess the trends for limbs of different sizes, which I suppose are the different colors. I request the authors to calculate the trends (with SEM) for limbs of different sizes independently. This will test the authors' conclusion that the position of the high-proliferation and high-density region are the same for limbs of different sizes.

7) I find that the Introduction is mainly based on the concept of allometry while it provides a minimal description of the role of the Shh/Fgf8 centers in axolotl limb regeneration. I request the authors to provide a more comprehensive Introduction to the role of these two pathways.

Minor points

8) The anterior-posterior axis has different directions in each image, which I find it confusing. I suggest the authors to rotate images such that the anterior-posterior axis is horizontal.

9) Line 120: I find the word "implied" too causal, given the correlative nature of the observations.

10) Line 188: "spatial" is typed "special".

11) Fig. 4O: I request the authors to show representative images related to these measurements.

First revision

Author response to reviewers' comments

Responses to the reviewers

Our comments to reviewers are written in red.

Reviewer 1 Comments for the Author:
Furukawa et al seek to understand the nature of the scaling laws that underlie the growth of limb blastema to achieve normal morphogenesis from different size start points. This is an important question which has seldom been tackled at the quantitative level and I commend the authors for doing so.

In a nutshell, the authors investigate the relationship between various parameters (blastema, stump or body size, Shh, Ptch1, FGF8 expression areas as well as relative A-P based sub-areas) and uncover important principles underlying blastema growth: that scaling is allometric, that the ratio of shh-fgf8 signaling-dominant regions is invariable, that Shh expression scales with blastema size, that maximum cell density & proliferation scale together with the Shh-Fgf8 signaling-dominant region, and provide a mathematical description for the growth scaling from digit formation to regenerating limb prior to secondary growth.
I find this work is highly relevant to the audience of Development, it is original, overall clearly written and discussed, and brings forward further research questions. As such I would encourage its publication. Nevertheless, there are a few suggestions and comments to consider ahead of this:

>>First of all, we are grateful for the positive and encouraging comments from the reviewer. We have revised our manuscript as thoroughly as possible. We hope our revisions adequately address the reviewer's requirements.

© 2024. Published by The Company of Biologists under the terms of the Creative Commons Attribution License (https://creativecommons.org/licenses/by/4.0/).

Reviewer 1

(1) The study addresses relationships which are applicable to blastema size yet all data derives from lower arm amputations. We know that there is a difference in blastema growth between upper and lower arm amputations. It appears from the text that the authors imply that the scaling laws would apply to upper arm blastemas as well, but it would be good to provide evidence that this is the case (at least regarding shh-fgf8 scaling), or else discuss it.

>>We appreciate the reviewer's feedback, which highlighted a potential misunderstanding regarding the applicability of our data to upper arm blastemas. We would like to clarify that we did not have any specific intention to apply the scaling laws derived from lower arm blastemas to upper arm blastemas. However, we attempted to seek the possibility that the reviewer mentioned. For this sake, we conducted additional experiments using several individuals, and the results are attached below (if the reviewer cannot find the attached file, please ask the editor for the PDF file we uploaded in the system). These experiments revealed that while some measurements from the upper limbs align with the allometric relationships derived from lower limb measurements, others do not conform to them very well. Regarding the data from upper arm amputations, we observed a trend in the allometric relationship, but more data points are needed to accurately quantify the coefficients. Due to time constraints, we decided not to pursue this further at this time. Instead, we have added the following statement to the Discussion section.

In the page 15, lines 342-347:
"Furthermore, the above-mentioned allometry and scale-invariance were derived from regeneration data following lower arm amputations. Since the blastema morphology and proliferation rate within lower arms are known to be different from the regeneration after upper arm amputations (Goss, 1969b; Iten and Bryant, 1973), comparing these data with the current results would be an interesting point for future research."

NOTE: We have removed unpublished data that had been provided for the referees in confidence.

(2) are the results from ectopic Shh expression (Fig 3A-C) corrected by electroporation efficiency?

>>No, they were not corrected by electroporation efficiency. Given the nature of electroporation, we consider that the number of plasmid copies introduced per cell and the number of cells receiving the plasmid vectors are uncontrollable, which makes the normalization difficult. Although we couldn't quantify such numbers, our results demonstrate that the ectopic expression of *Shh* expanded the gap size between the endogenous *Shh*(+) and *Fgf8*(+) with high reproducibility as shown in Figures 3B and C. Therefore, we concluded that the *Shh* and the *Fgf8* expressions are not independent of each other.

(3) Suppl Fig 4 U vs V : there is not only a shift in the proliferation peak but there appears to be an overall decrease or flattening of the proliferation profile overall – please discuss.

>>The vertical (Y) axis of Sup. Fig. 6U and V shows the relative value of BrdU/Hoechst signal density, normalized by dividing each value by the median for the respective sample, thus these graphs do not necessarily indicate an overall decrease in cell proliferation. Since the signal intensity varies in each sample, it is difficult to compare the absolute cell density and proliferation activity among samples. Regarding the flattening of the cell proliferation profile, it varied in each sample: Not all the samples showed the flattened cell proliferation profile. As shown in Revised Fig. 4N, even in the normal regeneration process, the cell proliferation distribution tends to vary more among individuals compared to the density distribution, although the clear reason for this is unknown. Therefore, it seems that flattening cannot be considered a highly reproducible phenomenon. Importantly, we, at least, confirmed that the region showing relatively higher cell proliferation activity expands anteriorly in the samples with ectopic *Shh* expression.

We revised the sentence in the page 11, lines 233-235, in order to clearly state the variation in the signal intensity among the samples.

Original version

© 2024. Published by The Company of Biologists under the terms of the Creative Commons Attribution License (https://creativecommons.org/licenses/by/4.0/).

"Median-normalized distributions were used for the comparison of cell density and proliferative activity among individuals with different sizes of blastema"
Revised version
"Since signal intensity varied across samples, we used median-normalized distributions for the comparison of cell density and proliferative activity among individuals with different sizes of blastema."

In accordance with the comment, we also added the sentences in the results section not in the Discussion section since we cannot find a proper position to mention it in the discussion section.
In P11, lines 245-247 Original version
"the area showing higher cell density and proliferative activity expanded to the anterior region (Sup. Fig. 4)."
Revised version
"the area showing higher cell density and proliferative activity appeared to be expanded to the anterior region, where the mitotic activity was relatively lower in the normal blastema (Fig. S6V, n = 4/5)."

(4) When the authors shift Shh domain by electroporation, and therefore the area of maximums density (as shown in Suppl Fig 4), do they shift also the position of Col2A expression?
>>In accordance with this comment, we performed experiments. Then, we noticed that the induction of ectopic *Shh* expression delayed the timing of digit differentiation, which made our analysis complicated. As far as we tested, *Col2a* expression representing digital cartilage did not appear in the blastema with ectopic *Shh* expression at the same stage as control samples showed *Col2a* expression (n = 5). The *Shh* expression level is known to decline as digit differentiation progresses. Therefore, it is possible that high *Shh* expression induced by the electroporation causes the suppression of the *Col2a* expression due to maintaining an undifferentiated state. Although we observed the samples with ectopic *Shh* expression at more time points, it was not easy to identify a time window where only the first appearing digit differentiated while the others did not, due to the alteration of the differentiation timing from the normal condition. Because of the long experimental time required for regeneration research, we couldn't identify this narrow time window this time. Therefore, we decided not to discuss the effect of ectopic *Shh* expression on the locations of cartilage condensation in this study.

(5) P. 7 line 142 'little correlated' should be changed to 'it does not correlate well'
>>We've made the changes as the reviewer suggested.

(6) P. 9 line 188 change 'special' to 'spatial'
>>We've made the changes as the reviewer suggested.

(7) Figure legends: check formatting of panels (bold vs normal)
>>We've made the changes as the reviewer suggested.

(8) Fig. 1: consistency of coefficient annotation (A vs B), add scales to D and E
>>Thank you for this comment. In Fig. 1A, the data were regressed to the allometry equation, i.e., $Y \propto X^a$, while in Fig. 1B, they were regressed to the constant function Y = const. because the data appeared to have a low dependence on the x-axis. Therefore, there is no issue with keeping the original figures as they are. Regarding the scale bars in Fig.1D and E, we've made the changes as the reviewer suggested.

Reviewer 2
I find this manuscript appropriate for the readership of Development. I find that the analysis and description of the methods should be strengthened in some parts of the manuscript. I propose to strengthen the mechanistic insights of this manuscript with an analysis and an experiment.
>>We appreciate the reviewer's recognition of the value in our manuscript. We have revised our manuscript as thoroughly as possible. We hope our revisions meet the reviewer's standard.

© 2024. Published by The Company of Biologists under the terms of the Creative Commons Attribution License (https://creativecommons.org/licenses/by/4.0/).

Major points

1) Most of the manuscript is based on the quantification of the sizes of the Shh-, Ptch1- and Fgf8-expressing domains by in situ hybridization. However, I find that the Methods describe vaguely how those domains are defined at the time of the analysis. I got the impression that they are manually selected, which can lead to biases. Given the importance of these measurements for the manuscript, I request the authors to design an automatic quantification method to define the sizes of Shh-, Ptch1- and Fgf8-expressing domains in each image.

   >>We re-measured gene expression domains in a semi-automatic manner. Concretely, we first binarized the image using the threshold tool on ImageJ (Fiji). Since the *in situ* hybridization signal intensity varied among samples, we set a threshold value for each sample. Automatic determination of the threshold was challenging, so the threshold values were manually set by visual inspection. Then, we removed auto-fluorescent signals mainly derived from blood cells manually. We illustrated this image processing method in the Materials and Methods section Fig. S2 in the revised version) and added explanations to the Materials and Methods section as follows.

   In P23, lines 504-512

   "The measurement of gene expression domains was made on binarized images (Fig. S2). We first rotated each image to make the A-P axis horizontal, then measured the A-P length of the blastema mesenchymal region. The image was binarized using the threshold tool in ImageJ (Fiji). Since the *in situ* hybridization signal intensity varied among samples, we set a threshold value for each sample. Automatic determination of the threshold was challenging, so the threshold values were manually set by visual inspection. We then removed auto-fluorescent signals, mainly derived from blood cells, manually. Finally, the coordinates of the anterior and the posterior ends of the binarized signals were obtained."

   >>As we modified the quantification method for the expression range as indicated above, this resulted in slight changes to the parameter values within the allometric equation for gene expression. Importantly, we confirmed that the change in the quantification method did not have any significant influence on our conclusion.

2) The authors switch between discussing sizes in absolute and relative terms across the manuscript, which I find confusing. I invite the authors to focus on the description in relative terms since it seems to be the most relevant for the results.

   >>Thank you for your valuable comment. After carefully reconsidering this point, we have decided to keep our sentences as it is. Since the scale-invariance on the relative sizes of the Shh/Fgf8-dominant region within the crosstalk region □SF was revealed using the allometric equation obtained by the measurement of absolute gene expression domain sizes, we consider it essential to show our results from both relative and absolute points of view.

3) Fig 3H, I. Here, the authors state that the ratio of the Shh-Ptch1 and Fgf8 domains is constant in limbs of different sizes. This is intuitive from Fig. 3H, but it is not as rigorously or as clearly reported as in other parts of the manuscript. I request the authors to report the result with a similar plot as in Fig. 3K

   >>We added a scatter plot in Fig. S5. The correlation coefficient between the relative size of the Shh signaling dominant region within the crosstalk region □SF and the blastema size was 0.216. This result confirms that the ratio of the Shh/Fgf8 signaling dominant region is almost scale invariant.

4) Fig. 3L-N. The authors propose a simple mathematical model to rationalize the observation that the ratio of the sizes of the Shh-Ptch1 and Fgf8 domains is constant. In essence, the authors say that the size of the region reached by the SHH protein does not scale with blastema size, because SHH diffusion and degradation do not. However, it is unclear how the Fgf8 domain would scale properly, besides from the fact of being downstream of SHH signaling. I suggest the authors to clarify this point.

   >>First, we want to clarify that the proposed mathematical model shows that the distance of the secreted SHH molecules travels from the anterior end of *Shh*(+) region is almost

© 2024. Published by The Company of Biologists under the terms of the Creative Commons Attribution License (https://creativecommons.org/licenses/by/4.0/).

independent of the blastema size, even if the size of *Shh*(+), the source size of secretion, increases roughly in proportion to the blastema size. Additionally, this mathematical model does not directly address the *Fgf8* expression pattern. Then, as the blastema size increases, the range of *Fgf8*(+) becomes relatively smaller (Fig. 2L, M). At this point, the detailed molecular mechanisms explaining this allometry remain unclear. As discussed in the original manuscript, the expression of *Fgf8* may be influenced by both activation and inhibition from the SHH signal. This is because if there were only activation effects, there would be no gap between *Shh*(+) and *Fgf8*(+). Therefore, investigating the detailed dose-response relationship of how *Fgf8* expression is determined by the intensity of the SHH signal will be crucial for understanding the allometry between the *Fgf8*(+) size and the blastema size. Furthermore, it is also possible that signals other than SHH contribute to the regulation of *Fgf8* expression. For example, in the limb buds of amniotes, it is known that the feedback loop between *Fgf8* and *Fgf10* is established independently from Shh signaling (Ohuchi et al., 1997), and it is reported that *Fgf10* is also expressed in axolotl limb bud and blastema (Christensen et al., 2002). In any case, as a fact, while the relative size of *Fgf8*(+) is allometrically dependent on the blastema size, within □SF, the relative size of *Fgf8*(+) remains approximately half regardless of the blastema size, which is one of our key findings.

references

>Ohuchi, H., Nakagawa, T., Yamamoto, A., Araga, A., Ohata, T., Ishimaru, Y., Yoshioka, H., Kuwana, T., Nohno, T., Yamasaki, M. et al. (1997) 'The mesenchymal factor, FGF10, initiates and maintains the outgrowth of the chick limb bud through interaction with FGF8, an apical ectodermal factor', Development 124(11): 2235-44.

>Christensen, R. N., Weinstein, M. and Tassava, R. A. (2002) 'Expression of fibroblast growth factors 4, 8, and 10 in limbs, flanks, and blastemas of Ambystoma', Dev Dyn 223(2): 193-203

Regarding the above points, we have revised both result and discussion section of the manuscript as follows:

In P10, lines 205-212 Original version

"However, since *Fgf8*(+) size depends on Shh signaling, its relative size also becomes smaller within larger blastemas probably due to the diffusion limit of SHH."

Revised version

"The relative size of *Fgf8*(+) also becomes smaller within larger blastemas. As demonstrated above, the expression of *Fgf8* depends on the Shh signal. In the context of limb development, it is also known that SHH activates *Fgf8* expression. However, considering the gap between *Shh*(+) and *Fgf8*(+), it is likely that there exists an inhibitory pathway, whether indirect or direct, in addition to the activation pathway. To quantitatively explain the allometric relationship between *Fgf8*(+) and blastema sizes (Fig. 2L-M), it would be necessary to elucidate the detailed dose- response relationship for *Fgf8* expression induced by the Shh signal."

We also added sentences in P17, lines 378-382 as follows:

"It is also possible that signals other than SHH contribute to the regulation of *Fgf8* expression. For example, in the limb buds of amniotes, the feedback loop between *Fgf8* and *Fgf10* is established independently from Shh signaling (Ohuchi et al., 1997), and it is reported that *Fgf10* is also expressed in the axolotl limb bud and blastema (Christensen et al., 2002)."

5) In Fig. 3, the authors show that the experimental over-expression of SHH shifted the position of the Fgf8-expressing domain.
   a) I request the authors to correlate the shift in position and change in size of the Fgf8 domain with the level of Shh-2a-GFP over-expression. I expect this measurement to help clarify the quantitative relationship between Shh and Fgf8 expression.
   >> Thank you for the comments. This comment is basically the same as reviewer 1's 2nd comment. Our response is as follows: "Given the nature of electroporation, we consider that the number of plasmid copies introduced per cell and the number of cells receiving the plasmid vectors are uncontrollable, which makes the normalization difficult. Although

© 2024. Published by The Company of Biologists under the terms of the Creative Commons Attribution License (https://creativecommons.org/licenses/by/4.0/).

we couldn't quantify such numbers, our results demonstrate that the ectopic expression of *Shh* expanded the gap size between the endogenous *Shh*(+) and *Fgf8*(+) with high reproducibility as shown in Figures 3B and C. Therefore, we concluded that the *Shh* and the *Fgf8* expressions are not independent of each other."

b) If technically possible, I request the authors to assess the relative positions of the cell proliferation and high-density regions, as well as that of second digit formation, following Shh-2a-GFP over-expression. Do they change? Are results compatible with the model proposed by the authors?
>>As for this query, in the original manuscript, we presented the spatial patterns of the cell density and cell proliferation activity in the blastema in which *Shh* was ectopically expressed (Fig. S6). The cell density and cell proliferation activity appeared to expand to the anterior region.
>>Regarding the position of the *Col2a* expression, the point is almost the same as reviewer 1, 4th comment. The following is our answer: "In accordance with this comment, we performed experiments. Then, we noticed that the induction of ectopic *Shh* expression delayed the timing of digit differentiation, which made our analysis complicated. As far as we tested, *Col2a* expression representing digital cartilage did not appear in the blastema with ectopic *Shh* expression at the same stage as control samples showed *Col2a* expression (n = 5). The *Shh* expression level is known to decline as digit differentiation progresses. Therefore, it is possible that high *Shh* expression induced by the electroporation causes the suppression of the *Col2a* expression due to maintaining an undifferentiated state. Although we observed the samples with ectopic *Shh* expression at more time points, it was not easy to identify a time window where only the first appearing digit differentiated while the others did not, due to the alteration of the differentiation timing from the normal condition. Because of the long experimental time required for regeneration research, we couldn't identify this narrow time window this time. Therefore, we decided not to discuss the effect of ectopic *Shh* expression on the locations of cartilage condensation in this study."

6) I find that the presentation of the data of Fig. 4M-N can be confusing, as we cannot assess the trends for limbs of different sizes, which I suppose are the different colors. I request the authors to calculate the trends (with SEM) for limbs of different sizes independently. This will test the authors' conclusion that the position of the high-proliferation and high-density region are the same for limbs of different sizes.
>>As the reviewer suggested, we have calculated individually the trends for blastemas of different sizes and have shown them with the lines in different colors (Fig. 4M and N of the revised version). For data for each blastema, we resampled at equal density along the AP axis and then performed regression using a Gaussian process. This resampling was done because the original data density along the AP axis was non-uniform, which can affect the accuracy of regression. In Fig. 4M/4N of the revised version, the black broken line represents the regression performed on the data from all individuals, and the gray area indicates the 95% confidence interval of the regression curve. The peak of the cell density was almost consistent in various sizes of the crosstalk region ¤SF. On the other hand, the position of the peak in cell proliferation activity within individual blastemas exhibited substantial variation. However, it still reached its maximum value on the anterior side from the center of ¤SF. Furthermore, the regression using cell proliferation activity data from all samples showed that the maximum value was located close to the peak of cell density (the black broken line in Fig. 4N), which closely matched the position of initial chondrogenic condensation shown in Fig 5E. Based on these results, we have revised the manuscript as follows.

In P12, lines 253-262 Original version
"The results revealed that their relative patterns within ΩSF were reproducible across individuals, independent of blastema size, and could be fitted well by a unimodal function (the black curves in Figs. 4M-N). In particular, cell density and proliferative activity peaked around the central region of the ΩSF (more precisely, a little anterior to the center, approximately 60% from the posterior end) which is thought to be more affected by both Shh and Fgf8 signaling (Fig.4N)."

© 2024. Published by The Company of Biologists under the terms of the Creative Commons Attribution License (https://creativecommons.org/licenses/by/4.0/).

Revised version
"The results revealed that their relative patterns of cell density within ΩSF were reproducible across individuals, independent of ΩSF (or blastema) size (Fig. 4M). Cell density peaked around the central region of the ΩSF (more precisely, a little anterior to the center, approximately 60% from the posterior end) in blastemas of all sizes we examined, which is thought to be more affected by both Shh and Fgf8 signaling. We further observed large variation in the distribution of cell proliferation among samples. However, the peak of cell proliferation activity still basically occurred a little anterior to the center. Notably, the peak position of the regression curve for the data from all samples closely matched that of the cell density distribution (the black broken curve in Fig. 4N)."

7) I find that the Introduction is mainly based on the concept of allometry, while it provides a minimal description of the role of the Shh/Fgf8 centers in axolotl limb regeneration. I request the authors to provide a more comprehensive Introduction to the role of these two pathways.
>>In accordance with the reviewer's comment, we have included additional information regarding *Shh* and *Fgf8* that is relevant to the content of this study in the Introduction as below.

In P3-4, lines 59-73
"*Shh* is expressed in the posterior region of the blastema during limb regeneration, as it is expressed in the developing limb buds of amniotes and axolotls (Torok et al., 1999b; Nacu et al., 2016; Purushothaman et al., 2019). However, *Fgf8* expression patterns in developing limb buds are significantly different between amniotes and urodele amphibians (Han et al., 2001; Nacu et al., 2016; Purushothaman et al., 2019). In amniotes' limb buds, *Fgf8* is expressed in an ectodermal region called the apical ectodermal ridge, while *Fgf8* is expressed in the anterior mesenchyme in axolotl limb buds. Although the *Fgf8* expression domain is different, a recent study suggests that the interaction between *Shh* and *Fgf8* in a developing limb bud is established in axolotls as it is in amniotes (Purushothaman et al., 2019). In axolotls, it has further been suggested that the Shh-Fgf8 feedback loop contributes to anteroposterior axis formation (Nacu et al., 2016). In the case of axolotl limb regeneration, the unique *Fgf8* expression pattern and interaction between *Shh* and *Fgf8* were recapitulated in a regenerating blastema, highlighting the similarities between the limb development and the limb regeneration processes."

Minor points

8) The anterior-posterior axis has different directions in each image, which I find it confusing. I suggest the authors to rotate images such that the anterior-posterior axis is horizontal.
>>In accordance with the reviewer's comment, we rotated the image in Fig.2(A), (B), (E), and (F). We also added some explanation in the Figure Legend

In P 34, lines 779-781
"The images in (A), (B), (E), and (F) were rotated to make the A-P axis horizontal. The blank regions caused by the rotation were filled in gray."

9) Line 120: I find the word "implied" too causal, given the correlative nature of the observations.
>>the word "implied" was replaced by "suggested"

10) Line 188: "spatial" is typed "special".
>>We've made the changes as you suggested.

11) Fig. 4O: I request the authors to show representative images related to these measurements.
>>We apologize for any misunderstandings caused by the way we presented the information. As shown in the Material and Method section, the data was obtained by quantitative real-time PCR. Thus, there is no representative images regarding this data.

Second decision letter

MS ID#: DEVELOP/2024/202697

© 2024. Published by The Company of Biologists under the terms of the Creative Commons Attribution License (https://creativecommons.org/licenses/by/4.0/).

MS TITLE: Allometry in limb regeneration and scale-invariant patterning as a basis of normal morphogenesis from different sizes of blastemas

AUTHORS: Saya Furukawa, Sakiya Yamamoto, Ayaka Ohashi, Yoshihiro Morishita, and Akira Satoh

I have now received all the referees reports on the above manuscript, and have reached a decision. The referees' comments are appended below, or you can access them online: please go to BenchPress and click on the 'Manuscripts with Decisions' queue in the Author Area.

The overall evaluation is positive and we would like to publish a revised manuscript in Development, provided that the referees' comments can be satisfactorily addressed. Reviewer #2 has comments that I think should be addressed, in particular because the intention is to improve rigor and data presentation. Please attend to all of the reviewers' comments in your revised manuscript and detail them in your point-by-point response. If you do not agree with any of their criticisms or suggestions explain clearly why this is so. If it would be helpful, you are welcome to contact us to discuss your revision in greater detail. Please send us a point-by-point response indicating your plans for addressing the referees' comments, and we will look over this and provide further guidance.

Reviewer 1

Advance summary and potential significance to field

Furukawa et al seek to understand the nature of the scaling laws that underlie the growth of limb blastema to achieve normal morphogenesis from different size start points. This is an important question which has seldom been tackled at the quantitative level and I commend the authors for doing so.

In a nutshell, the authors investigate the relationship between various parameters (blastema, stump or body size, Shh, Ptch1, FGF8 expression areas as well as relative A-P based sub-areas) and uncover important principles underlying blastema growth: that scaling is allometric, that the ratio of shh-fgf8 signaling-dominant regions is invariable, that Shh expression scales with blastema size, that maximum cell density & proliferation scale together with the Shh-Fgf8 signaling-dominant region. Further, they provide a mathematical description for the growth scaling from digit formation to regenerating limb prior to secondary growth.

Comments for the author

The authors have addressed my comments in an acceptable manner and thus I recommend this manuscript for publication.

Reviewer 2

Advance summary and potential significance to field

In this revised manuscript, the authors addressed most requests and suggestions of the previous review. They have improved and extended their analysis and text, as well as performed new experiments.

Comments for the author

In this revised manuscript, the authors addressed most requests and suggestions of the previous review. They have improved and extended their analysis and text, as well as performed new experiments.

The revised manuscript addressed most of my concerns. Following reading the revised manuscript and responses to the reviewers, I have a few additional suggestions/comments:

© 2024. Published by The Company of Biologists under the terms of the Creative Commons Attribution License (https://creativecommons.org/licenses/by/4.0/).

1) Reviewer 1 and I requested the authors to normalize the results of Shh-2a-GFP over-expression by the efficiency of Shh-2a-GFP electroporation (GFP expression). The authors say that Shh-2a-GFP electroporation efficiency varies widely and therefore this normalization cannot be performed. I do not find this explanation convincing, given that they likely have the GFP expression data and so they can indeed estimate electroporation efficiency. I invite the authors again to present an analysis showing the strength of the phenotype as a function of Shh-2a-GFP expression. If the analysis is not conclusive, I invite the author to elaborate on why that is the case.

2) The authors performed a new experiment evaluating the effect of Shh over-expression on the position of the second developing digit (Col2a expression). The experiment did not give an interpretable result, since Shh over-expression impaired digit formation. Although the experiment is not conclusive, it does provide information on the effects of Shh over-expression and I invite the authors to include it in the manuscript, if they have not already done so.

3) Fig 4M-N: I invite the author to use larger bins along the x-axis and average y-data in each bin. Resampling does not seem to me an appropriate procedure in this case.

4) FigS5: please report the P-value for the indicated correlation coefficient (null hypothesis: no correlation).

5) I suggest the authors to report P-values in each plot, instead of significance levels (asters).

6) I invite the authors to report the error in estimated parameters, for example fitted exponents, to a single significant digit and to round the estimated parameter accordingly. Similarly, P-values should be rounded to the first significant digit.

Second revision

Author response to reviewers' comments

Responses to the reviewers
Our comments to reviewers are written in red.

Reviewer 2 Comments for the Author:
In this revised manuscript, the authors addressed most requests and suggestions of the previous review. They have improved and extended their analysis and text, as well as performed new experiments.

The revised manuscript addressed most of my concerns. Following reading the revised manuscript and responses to the reviewers, I have a few additional suggestions/comments:

1)Reviewer 1 and I requested the authors to normalize the results of Shh-2a-GFP over-expression by the efficiency of Shh-2a-GFP electroporation (GFP expression). The authors say that Shh-2a-GFP electroporation efficiency varies widely and therefore this normalization cannot be performed. I do not find this explanation convincing, given that they likely have the GFP expression data and so they can indeed estimate electroporation efficiency. I invite the authors again to present an analysis showing the strength of the phenotype as a function of Shh-2a-GFP expression. If the analysis is not conclusive, I invite the author to elaborate on why that is the case.

>>Thank you for pointing out the importance of analyzing the relationship between the efficiency of ectopic *Shh* expression induction and the observed phenotypes. We agree that this is a very interesting and important topic to explore, as it could lead to a deeper understanding of how Shh signaling regulates the position of *Fgf8* expression. In this revision, we reconsidered the evaluation of how this ectopic *Shh* expression might influence the resulting gap size between endogenous *Shh*(+) and *Fgf8*(+). The pattern of the induced ectopic expression of *Shh*/GFP through electroporation can be characterized by a function S(x,y) that represents 'where' and 'how much'

© 2024. Published by The Company of Biologists under the terms of the Creative Commons Attribution License (https://creativecommons.org/licenses/by/4.0/).

the expression occurs, and this is thought to influence the *Shh-Fgf8* gap (δ). However, it is difficult to precisely control the location and level of induction by the electroporation, making it challenging to perform repeatable experiments with reproducible induction patterns. As a result, accurately elucidating the quantitative relationship between S(x,y) and the gap size δ between endogenous *Shh*(+) and *Fgf8*(+) regions is quite difficult. As one trial, we tested the total area of GFP-positive regions, S_{GFP+}, as a measure (or reduced information) of the strength of *Shh*/GFP induction, and examined its relationship with the gap size δ. The results showed no clear correlation between them (please see the revised Fig. S4). However, as mentioned above, due to the difficulty in achieving a reproducible induction pattern, this result of no clear correlation between S_{GFP+} and δ does not necessarily mean that there is no functional relationship between S(x,y) and δ. In fact, as shown in Fig. 3C, the mean gap size in the case of *Shh* induction is significantly larger compared to that of the control case, clearly showing the interaction between Shh and Fgf8 signaling. As detailed later, in the revised manuscript, we included a scatter plot of the GFP-positive area versus the *Shh-Fgf8* gap as a supplementary figure, noting that we did not observe no clear correlation between the GFP-positive area and the *Shh-Fgf8* gap. Next, we also considered an analysis based on the fluorescence intensity of the introduced GFP. In our experiment, GFP fluorescence was observed on sections after performing immunofluorescence staining for GFP (using a secondary antibody). This was because the native GFP fluorescence on the fifth day of electroporation was weak, probably due to the dilution of the plasmid through cell division and/or various experimental manipulations. Consequently, we judged that quantitative evaluation, especially through direct comparison of data between individuals, would be difficult. In summary, due to technical limitations, we were unable to draw quantitative conclusions about the effect of ectopic *Shh* expression induced by the electroporation method on the *Fgf8* expression pattern (location). However, we would like to emphasize that the results shown in this study (Figure 3A-C) are sufficient to support our claim that ectopic *Shh* expression influences the location of *Fgf8* expression. A more quantitative analysis is an important topic for understanding the mechanism by which stumps of different initial sizes achieve normal regeneration, and we hope to address this in the near future. Once again, we would like to express our sincere gratitude to the reviewers for their insightful comments.

We added the sentences as follows in lines 167-177:

It should be noted that while we are interested in the more quantitative regulatory relationship between *Shh* and *Fgf8* expression, it was difficult to evaluate this in the current experimental system. In the electroporation method, it is difficult to precisely control the location and level of expression induction, which made it challenging to perform repeatable experiments with reproducible induced patterns. As an exploratory trial, we examined the relationship between the total area of GFP(+) regions and the gap between endogenous *Shh*(+) and *Fgf8*(+), and we were unable to observe a clear correlation between them (Fig. S4) although the mean gap size in the case of *Shh* induction is significantly larger compared to that of the control case as seen in Fig. 3**C**. Clarifying the quantitative regulatory relationship between the spatial expression patterns of *Shh* and *Fgf8* will be an important issue for future research.

We also wrote the legend for Fig. S4 as follows:

The relationship between the total area of the GFP(+) regions and the gap size between the endogenous Shh(+) and Fgf8(+)
The vectors, pCS2-AcGFP or pCS2-Shh-p2a-AcGFP were electroporated into the blastema mesenchyme. The scatter plot shows the relationship between the total area of GFP(+) and the size of the endogenous Shh(+) and Fgf8(+) gap. The correlation coefficients R were 0.46 for pCS2-AcGFP EP (n = 12) and -0.13 for pCS2-Shh-p2a-AcGFP EP (n = 8), respectively.

2)The authors performed a new experiment evaluating the effect of Shh over-expression on the position of the second developing digit (Col2a expression). The experiment did not give an interpretable result, since Shh over-expression impaired digit formation. Although the experiment is not conclusive, it does provide information on the effects of Shh over-expression and I invite the authors to include it in the manuscript, if they have not already done so.

>>We appreciate the reviewer's recognition of the value of our additional experiments. In response to the comments, we have added the data as Fig. S8 to our manuscript, regarding the delayed

© 2024. Published by The Company of Biologists under the terms of the Creative Commons Attribution License (https://creativecommons.org/licenses/by/4.0/).

expression of *Col2* in the *Shh*-overexpressed blastemas. Additionally, we have included the following sentences in the main text in lines 313-321:

“We also examined how ectopic *Shh* expression affects the formation of digit II (Fig. S8). As a result, we found that the induction of ectopic *Shh* expression delayed the timing of digit differentiation, probably because Shh signaling maintains mesenchymal cells in an undifferentiated state. In the induction using the electroporation method, it is difficult to precisely control the location and amount of the ectopic expression, which likely caused variability in the degree of differentiation delay between samples. This made it difficult to determine the timing of the first digit formation and to quantitatively evaluate the location where the formation begins.”

We wrote the legend for Fig. S8 as follows:

The delay of the digit differentiation due to the ectopic induction of Shh expression
(A-H) The vectors, pCS2-AcGFP (A-D) or pCS2-Shh-p2a-AcGFP (E-H) were electroporated into the blastema mesenchyme. High magnification images of the squared region in A-H are shown in A’-H’. The construct was electroporated at a slightly later stage than in the case of Fig. 3C and Fig. S7. When the pCS2-AcGFP vector was introduced, the Col2-expressing cells showed an aggregation in most samples (n = 5/6) (A-D). However, when the pCS2-Shh-p2a-AcGFP was introduced, the concentrated expression pattern of Col2 was not observed; instead, its expression signals appeared sparsely throughout the entire mesenchymal region (n = 4/5) (E-H). Arrowheads in (E’) show ectopic Shh expression signals. The dotted curves indicate the boundary of the blastema epithelium. Scale bars in (A) and (E): 400μm, in (A’) and (E’): 200μm.

3)Fig 4M-N: I invite the author to use larger bins along the x-axis and average y-data in each bin. Resampling does not seem to me an appropriate procedure in this case.

>>Thank you for the valuable comment. According to this comment, we reanalyzed the data by using larger bins along the x-axis and average y-data in each bin (please see the revised Fig 4M-N).

4)FigS5: please report the P-value for the indicated correlation coefficient (null hypothesis: no correlation).

>>As the reviewer suggested, we conducted a correlation test (null hypothesis: no correlation). The obtained p-value was 0.2, and the null hypothesis was not rejected. This result means that the data do not support the existence of a correlation between the blastema size and the relative size of the Shh signaling dominant regions. Since we propose that the relative sizes of the Shh signaling dominant regions are scale-invariant, this statistical result is consistent with our claim.

We added the sentences explaining this result to the legend for Fig. S6 (Fig. S5 in the previous version) as follows:
The p-value for a correlation test (null hypothesis: no correlation) was 0.2, and the null hypothesis was not rejected.

5)I suggest the authors to report P-values in each plot, instead of significance levels (asters).

>>As the reviewer suggested, we reported P-values to one significant digit in each plot.

6)I invite the authors to report the error in estimated parameters, for example fitted exponents, to a single significant digit and to round the estimated parameter accordingly. Similarly, P-values should be rounded to the first significant digit.

>>As the reviewer suggested, we decided to report the error in estimated parameters to a single digit and to round the estimated parameter to the same decimal place, e.g. 0.90 ± 0.06, 0.299 ± 0.006.

© 2024. Published by The Company of Biologists under the terms of the Creative Commons Attribution License (https://creativecommons.org/licenses/by/4.0/).

Third decision letter

MS ID#: DEVELOP/2024/202697

MS TITLE: Allometry in limb regeneration and scale-invariant patterning as a basis of normal morphogenesis from different sizes of blastemas

AUTHORS: Saya Furukawa, Sakiya Yamamoto, Ayaka Ohashi, Yoshihiro Morishita, and Akira Satoh
ARTICLE TYPE: Research Article

Thank you for your patience. I am happy to tell you that your manuscript has been accepted for publication in Development, pending our standard publication integrity checks.

Reviewer 2

Advance summary and potential significance to field

The authors have addressed my requests, either with new experiments, analysis edits or discussions. Of note, the authors have assessed experimentally the relationship between Shh-2a-GFP over-expression and the gap size between the Shh/Fgf8 domains. The experiment gave a negative result, but the authors reported and discussed this outcome fairly. Overall, I think the manuscripts is now acceptable for publication.

Comments for the author

Additional point: I invite the authors to add P-value of correlation to the new Fig. S6 and to any other panel where it may be missing.

© 2024. Published by The Company of Biologists under the terms of the Creative Commons Attribution License (https://creativecommons.org/licenses/by/4.0/).
